# Contemporary comparative surgical outcomes of type A aortic dissection in US and China: an analysis of the national inpatient sample database and a Chinese multi-institutional registry

**DOI:** 10.1186/s13019-024-03023-z

**Published:** 2024-11-14

**Authors:** Feng Jiang, Xiaodi Wang, Michael Carmichael, Yanfei Chen, Ruijian Huang, Yue Xiao, Jifang Zhou, Cunhua Su

**Affiliations:** 1 School of International Business, China Pharmaceutical University, Nanjing, Jiangsu China; 2 Department of Thoracic and Cardiovascular Surgery, Nanjing First Hospital, Nanjing Medical University, Nanjing, Jiangsu China

**Keywords:** Cohort study, In-hospital mortality, Perioperative complication, Type a aortic dissection, Surgery

## Abstract

**Background:**

To investigate the contemporary comparative inpatient prognosis among US and Chinese patients with type A aortic dissection (TAAD).

**Methods:**

Data from Chinese multi-institutional TAAD registry and the US National Inpatient Sample databases were analyzed. We used multivariable logistic regression models to compare in-hospital mortality and perioperative complication rates between the US and China. Length of stay and overall costs were fitted with quantile regression models. Independent prognostic factors associated with post-operative survival were assessed via Cox proportional hazards models.

**Results:**

Among 3,121 eligible TAAD patients, 1,073 were from China (25.0% female; mean ± SD age, 53.9 ± 12.4) and 2,048 were from the US (31.2% female; mean ± SE age, 59.8 ± 0.3). During the study period, the in-hospital mortality rates in China and the US were 15.5% and 13.3%, yet the difference was insignificant after adjustment (aOR, 1.16; 95% CI, 0.69–1.97). While there was no significant difference in overall perioperative complications (aOR, 1.07; 95% CI, 0.52–2.18), the patterns of complications differed between two cohorts. While Chinese TAAD patients experienced significantly longer duration of hospitalization (median difference, + 10.4 days; 95% CI, 9.2–11.5), the US TAAD cohort had significantly greater overall hospitalization costs (49.9; 95% CI, 55.4–44.5, in 1000 USD).

**Conclusions:**

Notwithstanding significant differences in demographic and clinical characteristics, TAAD patients from China and the US demonstrated comparable in-hospital mortality and overall perioperative complication rates. Future initiatives should focus on expanding surgical eligibility to the elderly Chinese TAAD patients and optimizing the duration of hospitalization without undermining meaningful clinical outcomes.

**Trial registration:**

KY20220425-05, April 5th 25 2022.

**Supplementary Information:**

The online version contains supplementary material available at 10.1186/s13019-024-03023-z.

## Introduction

The burden posed by aortic dissection, specifically type A aortic dissection (TAAD), has been noteworthy due to its sudden onset, requiring immediate surgical interventions, and leading to substantial mortality rates. However, the mortality rate has been observed to vary significantly across different countries [1–4].

Indications for TAAD surgery as well as associate prognosis could vary across industrialized countries. In Japan, the incidence of operative mortality was found to be between 9 and 11%, [5, 6] while in the International Registry of Acute Aortic Dissection (IRAD), the rate was recorded to be as high as 18-25% [7]. In the United States, the mortality rate ranged from 15 to 20%, [8, 9]. while in Germany, it was 16.9% [10] and in the United Kingdom, the rate was between 15 and 23% [11, 12]. The differences in patient demographic and clinical characteristics, as well as the discrepancies in the provision of high-quality surgical services between countries, may have contributed to the unequal clinical outcomes between industrialized and middle-income countries such as China. Mortality rates for relevant studies in China in the same period ranged from 5 to 11%, and a higher proportion of younger patients with TAAD might partly explain the lower mortality rates [13–15]. Furthermore, the mortality rate has gradually decreased over the past decade due to better postoperative care and continuously improving surgical operations [4, 7–12]. Nevertheless, current data on comparative outcomes remained limited, especially during the period of disruption to life-saving surgical services worldwide caused by the COVID-19 pandemic [16].

The prevention and mitigation of perioperative complications were vital initiatives that aim to enhance the outcomes of type A open surgical repair (TASR). However, the available literature demonstrated a dearth of studies focusing on comparing the incidence rates and trends of perioperative complications across different nations [6–12]. Regrettably, current studies predominantly discussed the state of TAAD in specific geographic areas or nations, with the exception of the International Registry of Acute Aortic Dissection, which has not evaluated the specific conditions of TAAD patients in diverse countries [7, 17].

Therefore, the aim of this study was to compare the contemporary trends in in-hospital mortality and perioperative complications between US and Chinese patients with TAAD. To understand the shifting patterns of surgical management over time and to estimate the length of stay (LOS) and costs in real-world setting.

## Methods

### Data source

Data sources included the US National Inpatient Sample (NIS) databases (2015–2019) and inpatient data from multiple centers in China (2012–2021). At first, the Hospital Cost and Utilization Project’s consists of a series of databases and associated software tools, created by a federal-state-industry collaboration and funded by the Agency for Health Research and Quality. Within Hospital Cost and Utilization Project, the NIS serves as the largest publicly accessible claims database, capturing 20% of inpatient data across the United States.

On the other hand, Chinese multicenter data on TAAD were collected from 11 medical institutions in Jiangsu Province. A range of patient characteristics, including demographics, in-hospital mortality and related complications, and the type of operation performed were systematically recorded.

### Human ethics and consent to participate declarations

The experimental protocol was established, according to the ethical guidelines of the Helsinki Declaration and was approved by the Human Ethics Committee of the institutional review board of the China Pharmaceutical University. Since the data were deidentified, the requirement for informed consent was therefore waived.

### Patient population

The final analytical dataset consisted of 3,121 patients with TAAD who received surgical interventions during their index admission in the US and China during the study period. In the US cohort, we used a validated algorithm to determine types of aortic dissection [3, 8]. Patients received surgical management were categorized into three groups: type A open surgical repair (TASR), type B open surgical repair (TBSR) and thoracic endovascular aortic repair (TEVAR). We initially used ICD-9 to find procedure codes for dissection repair: resection of aorta, abdominal or thoracic vessels with replacement, resection of aorta or other thoracic vessels with anastomosis, or repair of blood vessel with synthetic patch graft or unspecified type of patch graft; procedure codes for cardiac surgery: cardioplegia, valve repair, or operations on vessels of the heart; and repeated the previous action using ICD-10. Patients with procedure codes for dissection repair and cardiac surgery were categorized as a TASR. Eligible patients were at least 18 years old at admission and had at least one ICD-9-CM or ICD-10-CM code indicative of aortic dissection. We subsequently excluded individuals from the study cohort who possessed unknown vital status at the time of discharge, exhibited indications of an aortic aneurysm, or underwent transfer on the day of admission. Relevant International Classification of Diseases codes were referred to in Supplemental Table 1.

In the Chinese TAAD cohort, patients with only endovascular or medical management or younger than 18 were excluded. We extracted relevant clinical variables from the patient registries of the hospitals, including patient demographics, clinical symptoms and signs, imaging findings, outcomes, and cause of death. We also continuously tracked the survival status and prognosis of some patients through telephone interviews.

### Outcomes measures

Our primary outcomes included in-hospital mortality and rates of complications. We also evaluated the length of hospitalization and healthcare costs for patients with TAAD in the United States and China. Further, we analyzed the surgical management patterns in the Chinese TAAD cohort and factors associated with long-term survival.

To directly compare the outcomes of Chinese and US patients, we used the demographic distributions extracted from the 2010 US census data to standardize the mortality and other indicators of the Chinese and US cohorts. Baseline characteristics were expressed as frequencies and percentages for categorical variables and medians (interquartile range, IQR) for continuous variables. Differences in baseline characteristics were compared by χ2 or rank sum test, as appropriate. Kaplan-Meier analysis was used to assess overall survival, and a multivariate Cox proportional hazards model was utilized to identify factors associated with overall mortality. All statistical tests were two-sided, with a *P* < 0.05 indicating statistical significance. Data were analyzed using R version 3.5.1 and SAS 9.4.

## Result

**Fig. 1:**
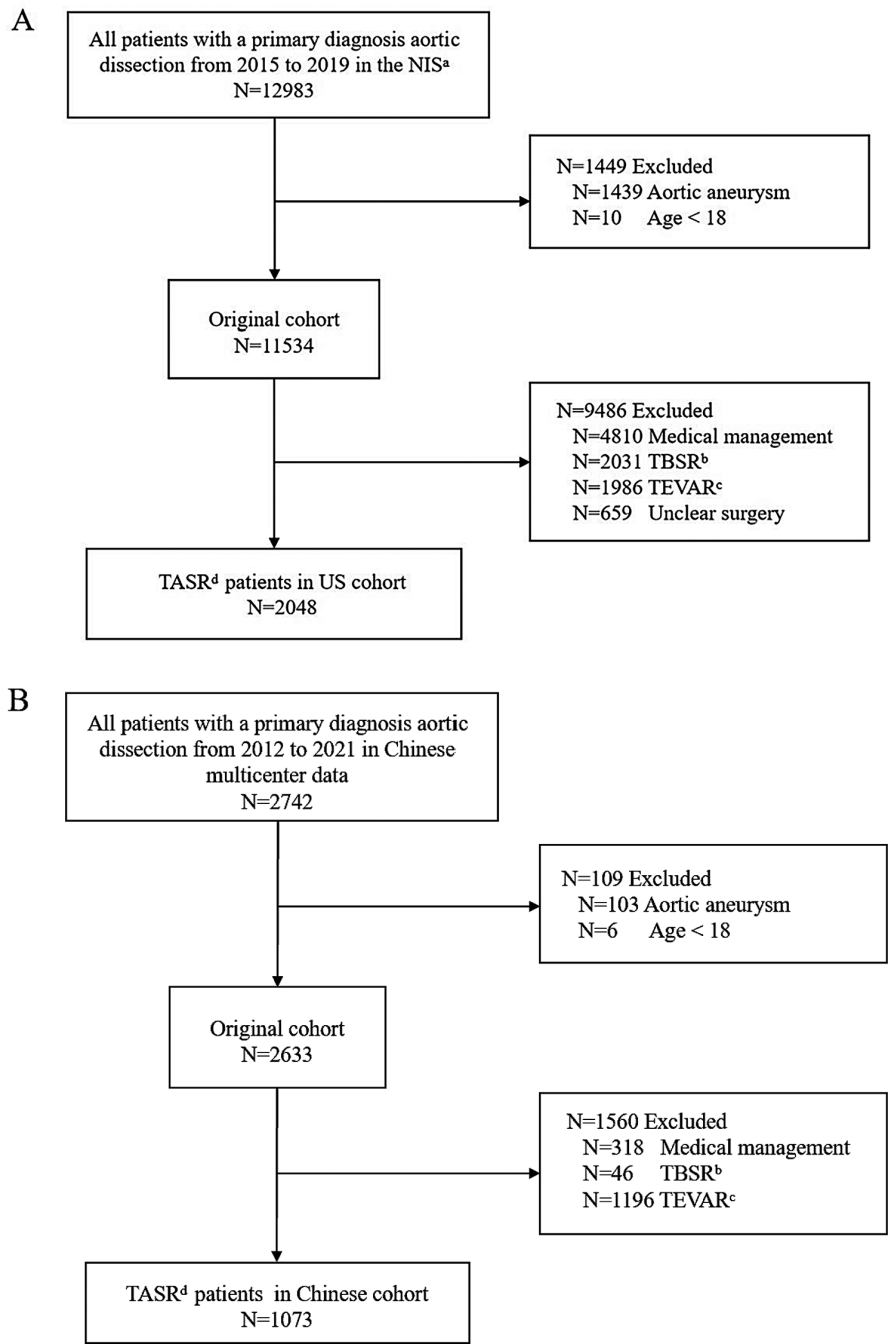
Study flow diagram of US (**A**) and Chinese (**B**) cohorts. Abbreviations: a NIS, National Inpatient Sample; b TBSR, type B open surgical repair; c TEVAR, thoracic endovascular aortic repair; d TASR, type A open surgical repair

The final cohorts included 3,121 inpatient admissions, with 2,048 from the United States and 1,073 from China (Fig. 1). In the Chinese multi-institution registry, patients were monitored post-discharge until death, loss to follow-up, or until the end of the study period (Dec 31st, 2021). the median (IQR) follow-up duration was 2.00 (1.58–2.75) years (Supplemental Fig. 1).

### Baseline characteristics

The baseline characteristics of the Chinese and US patients who underwent TAAD surgery throughout the study period were demonstrated in Table 1. The Chinese cohort was found to be younger, and had a lower proportion of women and higher weekend admissions than the US cohort (21.0% vs. 31.2%, *P* < 0.05; 28.5% vs. 23.1%, *P* < 0.05). Two cohorts also differed significantly in terms of racial status and insurance types. Notably, several comorbidities in the US cohort were more common than those in the Chinese cohort, such as type 2 diabetes, dyslipidemia, coronary artery disease and heart failure. On the other hand, a higher proportion of Chinese patients suffered from hypertension, chronic liver disease, stroke, and Marfan syndrome compared to the US counterparts. Overall, the prevalence rate of comorbidities was higher in the US cohort.

**Table 1:** Baseline descriptive demographic and clinical characteristics of the cohorts

	US NIS cohort (*N* = 2048)	Chinese cohort (*N* = 1073)	*P* value
**Age at admission**, (*yrs*, %*)*			
Mean (± SE/SD)	59.8 ± 0.3	53.9 ± 12.4	
Median (IQR)	60.3 (49.8–70.0)	54 (46–63)	
**Age group** (%)			< 0.0001
≤ 49	23.7	36.1	
50–59	23.5	30.4	
60–69	25.8	22.6	
70–79	20.5	10.0	
80+	6.5	0.9	
**Female sex** (%)	31.2	25.0	0.0003
**Year of admission** (%)			
2012–2015	22.5	22.6	
2016	18.4	6.7	
2017	19.6	8.4	
2018	18.3	10.1	
2019–2021	21.2	52.2	
**Race/ethnicity** (%)			< 0.0001
Non-Hispanic white	66.6	0	
African American	18.3	0	
Hispanic	7.3	0	
Asian, Pacific Islander	4.0	100	
Native American	0.2	0	
Other/unknown	3.6	0	
**Quartile of median income** (%)			
Quartile 1 (less wealthy)	26.0	NA	
Quartile 2	23.5	NA	
Quartile 3	24.9	NA	
Quartile 4 (wealthier)	25.8	NA	
**Insurance type** (%)			< 0.0001
Public insurance	54.0	54.3	
Private Insurance	36.0	0	
Self-pay/Others	10.0	45.7	
**Weekend admission** (%)	23.1	28.5	0.0009
**Chronic conditions** (%)			
Type 2 diabetes	10.9	7.2	0.0012
Hypertension	66.4	77.9	< 0.0001
Dyslipidemia	31.1	5.5	< 0.0001
Chronic kidney disease	5.8	5.7	0.91
Coronary artery disease	19.8	6.4	< 0.0001
Heart failure	20.4	14.1	< 0.0001
Chronic liver disease	1.2	5.7	< 0.0001
Stroke	4.9	6.7	< 0.0001
Chronic obstructive pulmonary disease	16.6	3.7	< 0.0001
Atrial fibrillation	23.8	4.2	< 0.0001
Obesity*	18.3	8.7	< 0.0001
Marfan syndrome	1.5	4.9	< 0.0001
**Charlson comorbidity index** (%)			< 0.0001
0–2	14.7	57.7	
3–4	31.2	32.4	
5+	54.1	9.9	

* Obesity status was determined using the estimated body mass index (BMI) at admission, with BMI equal or above 30 considered as obese

Abbreviations: NIS, national inpatient sample; SE standard error; SD, standard deviation; IQR, inter quartile range

### Mortality and perioperative complications

During the study period, in-hospital mortality for two cohorts decreased modestly, and the Chinese cohort seemed to have a better performance in this regard (Table 2**and** Table 3; OR, 0.84; 95% CI, 0.70-0.997). After accounting for greater mortality risks among the elderly patients, the standardized in-hospital mortality rates were comparable. Furthermore, mortality was not significantly different in the fully adjusted model (OR, 1.16; 95% CI, 0.69–1.97).

**Fig. 2:**
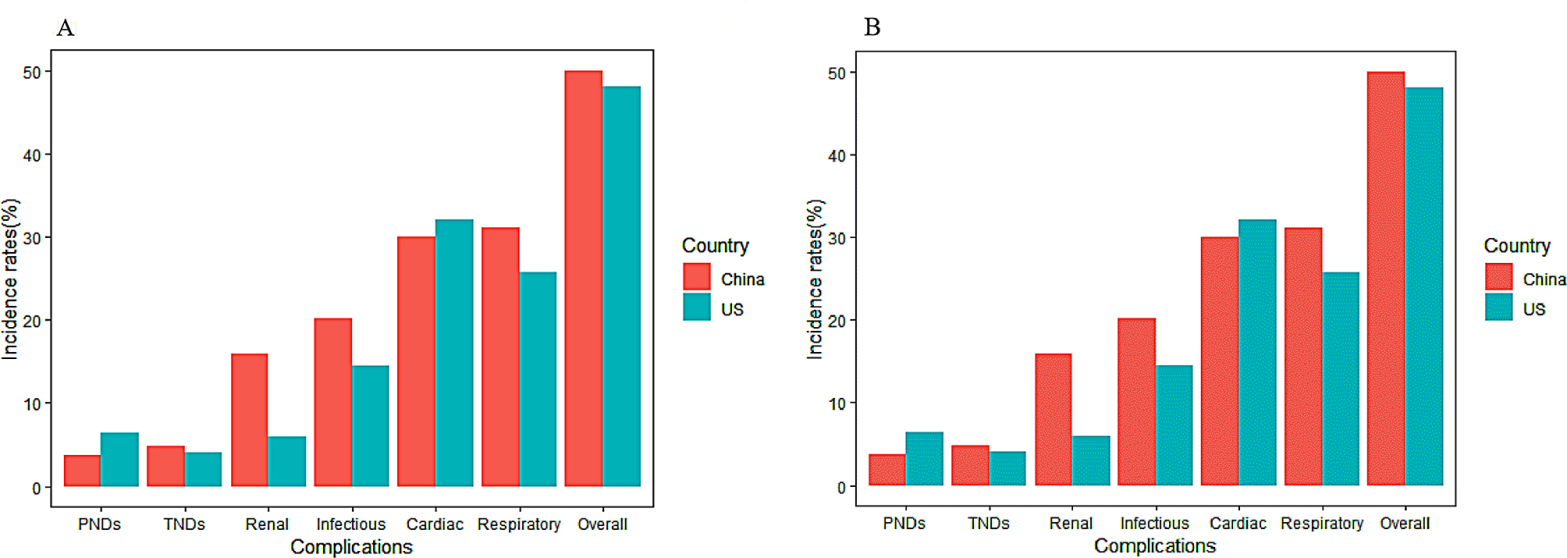
Comparison of crude and standardized incidence rates of perioperative complication between the Chinese and US cohorts. (**A**) Comparison of crude incidence rates of perioperative complication between the Chinese and US cohorts. (**B**) Comparison of standardized incidence rates of perioperative complication between the Chinese and US cohorts. Abbreviations: PNDS: Permanent neurological deficits; TNDS: Temporary neurological deficits

Renal complications were more prevalent in Chinese patients, and this finding was constant regardless of models (Fig. 2; Table 3; OR, 2.00; 95% CI, 1.25–3.19). Permanent neurological deficits were less common in the Chinese cohort than temporary neurological dysfunction, but the converse was true in the US cohort (OR, 0.51; 95% CI, 0.34–0.77; OR, 1.41; 95% CI, 1.01–1.98). The incidences of cardiac and respiratory complications were most prevalent, accounting for about half of all cases, and there was no significant difference between the US and Chinese cohorts (OR, 1.19; 95% CI, 0.77–1.85; OR, 0.61; 95% CI, 0.36–1.04). Similarly, there was no significant difference in overall complications (OR, 1.07; 95% CI, 0.52–2.18). Additionally, the trend of complication rates in the Chinese cohort showed greater volatility, while that in the US cohort was relatively stable (Supplemental Fig. 2).

**Table 2:** Observed crude and adjusted rates of in-hospital patient outcomes and associated costs in two cohorts

Outcomes	Overall	2012–2015	2016	2017	2018	2019–2021
Crude estimates (US)	(*N* = 2048)	(*N* = 460)	(*N* = 376)	(*N* = 402)	(*N* = 375)	(*N* = 435)
**in-hospital mortality**	15.5	17.4	17.6	12.9	15.2	14.5
in-hospital mortality(age:80+)	24.1	27.6	19.2	30.0	21.1	20.1
**LOS**,** in days (median**,** IQR)**	9.4 (6.0-15.7)	9.0 (5.8–14.4)	9.3 (6.2–15.4)	9.7 (6.0-15.4)	9.1 (5.6–16.6)	10.3 (6.2–17.2)
Prolonged stay*	7.5	5.4	8.8	5.5	7.5	10.6
**Waiting time**,** in days** (%)						
0	73.3	70.2	73.9	72.6	73.9	76.1
1	16.9	18.0	15.7	17.9	16.0	16.3
2+	7.6	11.7	10.4	9.5	10.1	7.6
**Costs**,** in1**,**000 US dollar (median**,** IQR)**	74.5 (51.0-112.7)	68.3 (50.0-99.8)	70.0 (50.9-109.1)	74.2 (51.6-110.5)	75.4 (49.6-109.8)	83.7 (54.1-134.3)
**Complications**						
Respiratory complications ^a^	43.5	48.9	40.4	42.0	46.1	39.3
Cardiac complications ^b^	53.7	59.8	52.1	52.5	53.9	49.7
Renal complications ^c^	9.3	8.5	10.1	8.0	10.4	9.9
Permanent neurological deficits ^d^	11.3	18.0	6.1	9.0	9.9	12.0
Temporary neurological dysfunction ^e^	7.6	5.7	5.3	8.7	8.0	10.1
Infectious complications ^f^	25.2	25.0	25.3	25.4	24.3	26.2
Overall complications	77.3	80.7	76.1	76.1	78.1	75.2
**Crude estimates (China)**	**(** ***N*** ** = 1073)**	**(** ***N*** ** = 243)**	**(** ***N*** ** = 72)**	**(** *N* ** = 90)**	**(** *N* ** = 108)**	**(** *N* ** = 560)**
**in-hospital mortality**	13.3	14.8	11.1	12.2	16.7	12.5
**LOS**,** in days (median**,** IQR)**	20 (16–26)	21 (17–26)	21.5 (19-25.5)	21.5 (18–28)	20 (15–24)	20 (15–27)
Prolonged stay*	17.2	18.9	13.9	18.9	9.3	18.0
**Waiting time**,** in days** (%)						
0	57.9	38.7	52.8	60.0	67.6	64.8
1	24.7	30.5	27.8	28.9	22.2	21.6
2+	17.4	30.9	19.4	11.1	10.2	13.6
**Costs**,** in 1**,**000 US dollar** **(median**,** IQR)**	31.1 (27.4–36.9)	29.6 (26.4–33.4)	30.1 (27.7–36.9)	33.4 (29.8–40.2)	32.6 (28.5–36.7)	31.2 (27.4–39.2)
**Complications**						
Respiratory complications ^a^	47.8	51.0	55.6	60.0	44.9	44.0
Cardiac complications ^b^	41.5	22.6	37.5	42.2	36.5	51.3
Renal complications ^c^	17.0	14.0	25.0	28.9	23.4	14.2
Permanent neurological deficits ^d^	6.1	7.4	5.6	6.7	9.4	4.5
Temporary neurological dysfunction ^e^	10.3	16.5	11.1	13.3	9.4	6.4
Infectious complications ^f^	28.1	32.5	29.2	13.3	23.4	27.8
Overall complications	68.7	65.4	76.4	71.1	63.9	69.8
**Age- and sex- standardized estimates (US)**						
**in-hospital mortality**	9.0	11.9	9.8	7.1	6.1	9.3
Prolonged stay*	4.2	3.2	5.8	3.0	4.8	4.8
**Complications**						
Respiratory complications ^a^	25.8	30.4	25.8	22.6	31.2	22.0
Cardiac complications ^b^	32.0	32.7	28.1	29.4	30.6	35.6
Renal complications ^c^	5.9	6.7	6.7	4.0	6.7	5.4
Permanent neurological deficits ^d^	6.5	10.9	3.1	4.0	7.1	8.3
Temporary neurological dysfunction ^e^	4.1	3.3	2.5	5.1	4.0	4.6
Infectious complications ^f^	14.5	14.7	13.6	15.0	14.9	15.8
Overall complications	48.1	49.1	45.6	44.8	49.4	50.7
**Age- and sex- standardized estimates (China)**						
**in-hospital mortality**	11.0	11.6	5.9	5.7	10.3	9.3
Prolonged stay*	16.3	13.7	5.8	9.9	5.9	19.4
**Complications**						
Respiratory complications ^a^	31.1	36.5	31.1	30.6	22.1	28.0
Cardiac complications ^b^	30.0	18.9	26.1	19.2	20.8	32.8
Renal complications ^c^	16.0	9.6	3.8	14.7	12.2	6.1
Permanent neurological deficits ^d^	3.8	6.1	3.0	1.7	3.3	3.5
Temporary neurological dysfunction ^e^	4.9	8.9	4.0	5.5	3.7	2.1
Infectious complications ^f^	20.2	26.1	16.7	13.0	13.7	17.5
Overall complications	50.0	53.2	45.1	37.6	33.5	45.8

*Prolonged stay was defined as admissions with length of stay equal or above 30 days

a Respiratory complications included hypoxia, pneumonia, prolonged ventilation (> 12 h), postoperative reintubation, acute respiratory distress syndrome and respiratory failure

b Cardiac complications included low cardiac output syndrome, cardiac arrest, postoperative arrhythmia, heart conduction disturbance, perioperative myocardial infarction, mitral insufficiency, aortic insufficiency, tricuspid insufficiency, perivalvular leakage, pericardial effusion and cardiac tamponade

c Renal complications included acute kidney injury and renal failure requiring renal replacement therapy

d Permanent neurological deficits included stroke, paraplegia, spinal cord complications and sensory disorders

e Temporary neurologic dysfunction included transient ischemic attack and delirium

f Infectious complications included bacteremia, sepsis, wound infection, infections of mediastinum, pulmonary infection/pneumonia, urinary tract infection, endocarditis, pericarditis; skin/soft tissue infection and infection of unknown site

Abbreviations: IQR, inter quartile range; LOS, length of stay

**Table 3:** Comparative outcomes between two cohorts on in-hospital mortality, prolonged length of stay and perioperative complications

Outcomes	China (Comparator, %)	US (Reference, %)	Absolute differences (95% CI)	OR (95% CI)
**Crude Model**				
In-hospital mortality	13.3%	15.5%	-0.02 (-0.05, 0.004)	0.84 (0.70, 0.997)
Prolonged stay*	17.2%	7.5%	0.10 (0.07, 0.12)	2.55 (1.88, 3.45)
Any perioperative complications	68.7%	77.3%	-0.09 (-0.12, -0.05)	0.65 (0.42, 1.01)
**Specific complications**				
Respiratory complications ^a^	47.8%	43.5%	0.04 (0.002, 0.08)	1.19 (0.77, 1.85)
Cardiac complications ^b^	41.5%	53.7%	-0.13 (-0.16, -0.09)	0.61 (0.36, 1.04)
Renal complications ^c^	17.0%	9.3%	0.08 (0.05, 0.10)	2.00 (1.25, 3.19)
Permanent neurological deficits ^d^	6.1%	11.3%	-0.06 (-0.08, -0.04)	0.51 (0.34, 0.77)
Temporary neurological dysfunction ^e^	10.3%	7.6%	0.02 (-0.01, 0.04)	1.41 (1.01, 1.98)
Infectious complications ^f^	28.1%	25.2%	0.03 (0.01, 0.06)	1.16 (0.93, 1.45)
Adjusted Model 1**				
In-hospital mortality	11.0%	9.0%		1.00 (0.81, 1.23)
Prolonged stay*	4.2%	16.3%		2.61 (1.93, 3.53)
Any perioperative complications	48.1%	50.0%		0.76 (0.48, 1.22)
Adjusted Model 2**				
In-hospital mortality	11.0%	9.0%		1.06 (0.74, 1.53)
Prolonged stay*	4.2%	16.3%		1.78 (1.20, 2.65)
Any perioperative complications	48.1%	50.0%		0.75 (0.45, 1.27)
Adjusted Model 3**				
in-hospital mortality	11.0%	9.0%		1.10 (0.74, 1.63)
Prolonged stay*	4.2%	16.3%		2.18 (1.38, 3.44)
Any perioperative complications	48.1%	50.0%		1.10 (0.56, 2.14)
Adjusted Model 4**				
in-hospital mortality	11.0%	9.0%		1.16 (0.69, 1.98)
Prolonged stay*	4.2%	16.3%		2.42 (1.29, 4.56)
Any perioperative complications	48.1%	50.0%		1.07 (0.52, 2.18)

*Prolonged stay was defined as admissions with length of stay equal or above 30 days

**Model 1 was adjusted for sex and age at admission; Model 2 was adjusted for sex, age, and race/ethnicity status; Model 3 was adjusted for all demographic and clinical characteristics; Model 4 was adjusted for all demographic and clinical characteristics and characteristics of healthcare facilities

a Respiratory complications included hypoxia, pneumonia, prolonged ventilation (> 12 h), postoperative reintubation, acute respiratory distress syndrome and respiratory failure

b Cardiac complications included low cardiac output syndrome, cardiac arrest, postoperative arrhythmia, heart conduction disturbance, perioperative myocardial infarction, mitral insufficiency, aortic insufficiency, tricuspid insufficiency, perivalvular leakage, pericardial effusion and cardiac tamponade

c Renal complications included acute kidney injury and renal failure requiring renal replacement therapy

d Permanent neurological deficits included stroke, paraplegia, spinal cord complications and sensory disorders

e Temporary neurologic dysfunction included transient ischemic attack and delirium

f Infectious complications included bacteremia, sepsis, wound infection, infections of mediastinum, pulmonary infection/pneumonia, urinary tract infection, endocarditis, pericarditis; skin/soft tissue infection and infection of unknown site

Abbreviations: OR, odds ratio

### Length of stay and health care cost

After comparing length of stay and costs between the Chinese and US cohorts, it was found that the Chinese cohort maintained a longer length of stay and lower total costs (Table 2). After adjusting for relevant covariates including demographic and clinical characteristics, such as age, sex, preoperative comorbid conditions, the Chinese cohort still had lower overall in-hospital costs (Table 4; median net increase, -49.9; 95% CI, -55.4–44.5). We further analyzed the Chinese cohort and found that patients who underwent total arch replacement surgery spent significantly more during their hospitalization than those who underwent ascending or hemi-arch replacement surgery (Supplemental Table 5). Meanwhile, the length of stay in the Chinese cohort was significantly longer than that in the US cohort (median net increase, 10.4; 95% CI, 9.2–11.5). Furthermore, we found the same tendency in prolonged stay (OR, 2.42; 95% CI, 1.29–4.56). On the other hand, related medical expenses in the United States continued to grow, while those in China remained stable during the study period. Additionally, the US cohort tended to have a shorter waiting time from admission to surgical procedures.

**Table 4:** Comparative outcomes between two cohorts on length of stay and overall costs

Outcomes	China (Comparator)	US (Reference)	Median Net Increase in LOS/Cost (Days/$) (95% CI)
**Quantile regression model**			
**Crude Model**			
LOS (median, IQR)	20 (16–26)	9.4 (6.0-15.7)	10.0 (10.0-32.3)
Costs, in 1,000 US dollar (median, IQR)	31.1 (27.4–36.9)	74.5 (51.0-112.7)	-43.4 (-44.3, -42.3)
Adjusted Model 1**			
LOS (median, IQR)	20 (16–26)	9.4 (6.0-15.7)	7.1 (5.2–9.4)
Costs, in 1,000 US dollar (median, IQR)	31.1 (27.4–36.9)	74.5 (51.0-112.7)	-42.4 (-43.3, -41.5)
Adjusted Model 2**			
LOS (median, IQR)	20 (16–26)	9.4 (6.0-15.7)	10.2 (9.3–11.6)
Costs, in 1,000 US dollar (median, IQR)	31.1 (27.4–36.9)	74.5 (51.0-112.7)	-51.9 (-54.3, -50.2)
Adjusted Model 3**			
LOS (median, IQR)	20 (16–26)	9.4 (6.0-15.7)	10.8 (9.7–11.6)
Costs, in 1,000 US dollar (median, IQR)	31.1 (27.4–36.9)	74.5 (51.0-112.7)	-45.3 (-48.0, -40.2)
Adjusted Model 4**			
LOS (median, IQR)	20 (16–26)	9.4 (6.0-15.7)	10.4 (9.2–11.5)
Costs, in 1,000 US dollar (median, IQR)	31.1 (27.4–36.9)	74.5 (51.0-112.7)	-49.9 (-55.4, -44.5)

*Prolonged stay was defined as admissions with length of stay equal or above 30 days

**Model 1 was adjusted for sex and age at admission; Model 2 was adjusted for sex, age, and race/ethnicity status; Model 3 was adjusted for all demographic characteristics; Model 4 was adjusted for all demographic and clinical characteristics and characteristics of healthcare facilities

Abbreviations: IQR, inter quartile range; LOS, length of stay

### Long-term survival

**Fig. 3:**
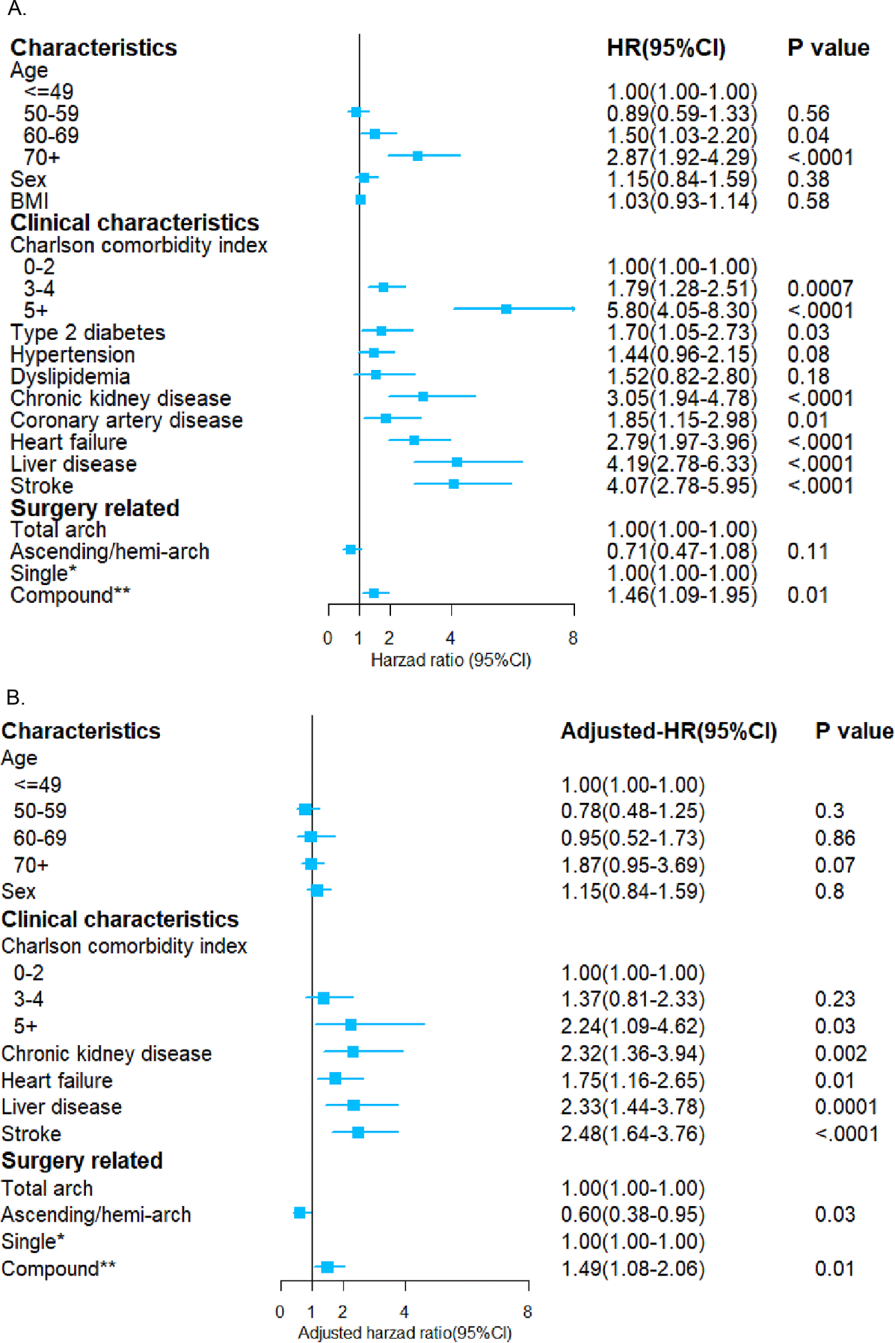
Unadjusted (**A**) and adjusted (**B**) predictive factors associated with overall survival in Chinese type A aortic Dissection cohort. *Only total arch or ascending/ hemi-arch were used in TAAD patients. **TAAD patients were treated with total arch or ascending/hemi-arch in combination with at least one of the following operations: Aortic valve replacement, Root replacement, Aortic valvuloplasty, Coronary artery bypass grafting, Bypass surgery, Mitral valve replacement or plasty and Pacemaker implantation

The preliminary results showed that age, burden of comorbidity (measured by Charlson comorbidity index), type 2 diabetes, chronic kidney disease, coronary heart disease, heart failure, stroke, liver disease and surgical complexity were all significantly related to the long-term survival of TAAD patients after surgery (Fig. 3, Supplemental Fig. 3 and Supplemental Table 3). For example, compared with patients under 50 years old, the long-term mortality rate of patients over 70 years old increased by 187% (HR, 2.87; 95% CI, 1.92–4.29). After adjusting the relevant variables, and the type and complexity of surgery still had a significant impact on the long-term survival rate. Among them, the choice of total arch replacement surgery or compound surgery was associated with lower long-term survival rate. We further investigated operative management in the Chinese cohort (Supplemental Table 4). Among analyzed TAAD patients, 80.7% underwent total arch replacement surgery, while 32.8% subjects underwent two or more types of surgical procedures.

## Discussion

In this study, we compared the characteristics and outcomes of patients undergoing surgery for TAAD by using the US NIS database and the Chinese multicenter registry. The Chinese cohort represented the largest contemporary Chinese TAAD cohort to undergo surgical management [13, 15, 18], and we collected follow-up data to analyze prognostic factors associated with long-term survival. As previously reported, Chinese patients were relatively younger and appeared to have lower comorbid burdens [13–15, 18]. The in-hospital mortality rates observed in US and Chinese patients were consistent with recent literature [8–10, 12, 19]. Nevertheless, other cohorts showed higher or lower mortality rates owing to differences in study period, country and demographic characteristics [6, 7, 11, 13–15, 18]. The waiting time from admission to surgery was significantly longer among the Chinese cohort compared to the US. Meanwhile, despite the longer length of stay in China, overall costs were lower compared to US hospitalizations for TAAD.

Mortality rates in the US and Chinese cohorts remained stable during the study period, while differences between the two countries persisted. There were five possible explanations for the difference in mortality rates before standardized. Firstly, the age distribution of the patients differed across the two nations. Specifically, elderly patients over the age of 80 accounted for only 0.9% of the Chinese cohort while composing 6.5% of the US cohort. Given the heightened mortality risk for individuals in this age group [9, 19, 20], questions remained unresolved regarding whether surgical intervention would benefit elderly patients with acute TAAD [21–24]. In China, considering that the reported in-hospital mortality and postoperative complications were both very high for patients aged above 80 years old at diagnosis, octogenarians with acute TAAD were more likely to defer surgery due to perceived worsened survival relative to medical management [22, 24] Secondly, most of the patients in the Chinese cohort were transferred to hospitals with greater accreditation for treatment, and prior studies have proven the mortality rate of TAAD patients was directly proportional to the hospital surgery volume [25]. Thirdly, many patients with acute TAAD might die before admission or diagnosis in China, owing to insufficient emergency rescue forces [14] Fourth, although no data was available from the NIS, according to a relatively new study from the University of Michigan, hemiarch replacement was 57.8% compared to aggressive arch replacement [26] Conversely, most Chinese surgeons thought that total arch replacement combined with stented elephant trunk implantation was a “standard” therapy for TAAD involving the repair of the aortic arch, which was 70.8% (80% in this study) compared to conventional surgical repair [13].

The present study found comparable rates of complications between the United States and China, yet the US cohort demonstrated a more consistent long-term trend in the occurrence of various complications. This finding might reflect the greater maturity of treatment strategies and overall healthcare delivery in the United States. On the contrary, the incidence rates of complications fluctuated greatly in Chinese patients, notably the rate of cardiac complications, which might be ascribed to an increase in the complexity of operations performed and the number of patients undergoing surgery for TAAD related to the establishment of Chest Pain Center. With increasing numbers of Chest pain Centers established across Jiangsu Province, the accessibility of emergency surgical services for local patients with complex TAAD has been drastically improved.

Our study corroborated that the US centers had higher turn-over rates as demonstrated by significantly shorter length of stay. Nevertheless, US centers charged for much higher medical expenses. The reasons for the difference in length of stay were complex and multifactorial. One reason was that the length of stay of patients was strictly limited by insurance companies in the US, while the cost was paid by basic medical insurance or out-of-pocket in China. Another reason was that discharged patients had the option of being transferred to post-acute care settings in the US, such as skilled nursing facility, rehabilitation or nursing home [27], which was quite different from the vast majority of Chinese patients returning home after discharge. While the most likely reason for the difference in overall costs could have been inexpensive surgical treatment and lower administrative expenditures in Chinese public health system.

The results of multivariate analysis indicated that the severity of associated complications and the choice of TAAD surgical methods were related to the long-term survival of patients, and the impact of age on that was not significant after variable adjustment. Bojko et al. reported that heart failure, postoperative renal failure and stroke were significant independent predictors of all-cause mortality, paralleling the results of the current study [28]. We also found total arch replacement or single surgery tended to be associated with reduced long-term survival. Nevertheless, the effects of different aortic dissection operations were controversial internationally [29]. The sample size of patients with ascending aortic replacement or hemiarch replacement patients may have limited the statistical power to detect disparities. Furthermore, the choice of surgical strategy depended on the patient’s condition and was not randomized. Therefore, further research was needed to prove the superiority of total arch replacement or hemiarch replacement. Additionally, our study revealed that advanced age did not exhibit a statistically significant correlation with long-term survival, which was consistent with previous studies on the surgical repair of acute TAAD in elderly patients [30].

This study provided valuable insights into potential avenues for enhancing the quality of care and outcomes for patients with TAAD in low- and middle-income countries such as China. Firstly, the medical institution will need to expedite the scheduling of emergency surgery and better coordinate patient transfer since TAAD is a life-threatening condition and emergency services are available mostly in major cities. Secondly, local medical institutions should start building emergency rescue capacities to ensure that patients receive timely rescue and proper medical management, and more specialized surgeons will need to be trained to manage an increasingly elderly and acute TAAD population.

In addition, limitations of the present study should be acknowledged. Firstly, there was heterogeneity in the Chinese and US databases. The NIS database lacked detailed patient medical information to further compare laboratory indicators, surgical procedures, etc. Moreover, Chinese patient data were limited to one province and represented challenges in terms of representativeness. Therefore, some of the findings presented in this paper should be interpreted with caution and considered within the context of diverse administrative frameworks.Secondly, the identification of patients with TAAD in the NIS was based on ICD-9 and ICD-10 codes, with pre-existing algorithms on the former converted to the latter. Although there were no abnormal fluctuations in the number of patients over the years, the accuracy and sensitivity of identification still needed further verification. In addition, the study duration was not the same for the US and Chinese cohorts, with the Chinese cohort having a longer study duration. This was mainly limited by the availability of data in the US.

## Conclusion

TAAD patients in China were approximately five years younger compared to those in the US. There seemed to be no significant difference between the Chinese and US cohorts in terms of in-hospital mortality and overall perioperative complications. We revealed variations in length of stay and cost, underscoring the unique characteristics of patients with TAAD receiving care within respective healthcare system.

## Electronic supplementary material

Below is the link to the electronic supplementary material.

Supplementary Material 1

## Data Availability

No datasets were generated or analysed during the current study.

## Abbreviations

TAAD: Type A aortic dissection
SD: Standard deviation
SE: Standard error
Aor: Adjusted odds ratio
CI: Confidence interval
IRAD: International Registry of Acute Aortic Dissection
TASR: Type A open surgical repair
LOS: Length of stay
NIS: National Inpatient Sample
HCUP: Hospital Cost and Utilization Project
TBSR: Type B open surgical repair
TEVAR: Thoracic endovascular aortic repair
ICD-9: International Classification of Diseases, Ninth Revision
IQR: Interquartile range
OR: Odds ratio
HR: Hazard ratio
