# Supplementary material for: Contemporary comparative surgical outcomes of type A aortic dissection in US and China: an analysis of the national inpatient sample database and a Chinese multi-institutional registry
Source: J Cardiothorac Surg. 2024 Nov 14;19:632. doi: 10.1186/s13019-024-03023-z (PMC11566593; doi:10.1186/s13019-024-03023-z)
Supplement: Supplementary file 1 — Supplementary Material 1 [file 13019_2024_3023_MOESM1_ESM.docx]

Supplemental Table 1. Diagnosis and procedure codes used in the present study in condition ascertainment

Supplemental Table 2. Descriptive characteristics of healthcare facilities treating acute type A aortic dissection in two cohorts

Supplemental Table 3. Predictive factors associated with overall survival in Chinese cohort of type A aortic dissection

Supplemental Table 4. Descriptive characteristics of surgical types of acute type A aortic dissection in Chinese cohorts

Supplemental Table 5. Impact of different surgical types on outcomes in Chinese cohorts

Supplemental Figure 1. Survival plot on long-term all-cause mortality in the Chinese cohort

Supplemental Figure 2. Crude and standardized complication trend charts for the Chinese and US cohorts

Supplemental Figure 3. Survival plot for subgroups of long-term all-cause mortality in the Chinese cohort

**Supplemental Table 1.** Diagnosis and procedure codes used in the present study in condition ascertainment

| **Projects** | **ICD-9 codes** | **ICD-10 codes** |
| --- | --- | --- |
| Thoracic aortic dissections | 441.01 | I71.01 |
| Dissection of thoracoabdominal aorta | 441.03 | I71.03 |
| Aortic aneurysms | 441.1-441.7,441.9 | I71.1-I71.6, I71.8, I71.9 |
| Dissection repair |  |  |
| Resection of aorta, abdominal or thoracic vessels with replacement | 38.44-38.45 | 04R007Z,04R00JZ,04R00KZ,04R047Z,04R04JZ,04R04KZ,02RP07Z,02RP08Z,02RP0JZ,02RP0KZ,02RP47Z,02RP48Z,02RP4JZ,02RP4KZ,02RQ07Z,02RQ08Z,02RQ0JZ,02RQ0KZ,02RQ47Z,02RQ48Z,02RQ4JZ,02RQ4KZ,02RR07Z,02RR08Z,02RR0JZ,02RR0KZ,02RR47Z,02RR48Z,02RR4JZ,02RR4KZ,02RS07Z,02RS08Z,02RS0JZ,02RS0KZ,02RS47Z,02RS48Z,02RS4JZ,02RS4KZ,02RT07Z,02RT08Z,02RT0JZ,02RT0KZ,02RT47Z,02RT48Z,02RT4JZ,02RT4KZ,02RV07Z,02RV08Z,02RV0JZ,02RV0KZ,02RV47Z,02RV48Z,02RV4JZ,02RV4KZ,02RW07Z,02RW08Z,02RW0JZ,02RW0KZ,02RW47Z,02RW48Z,02RW4JZ,02RW4KZ,02RX07Z,02RX08Z,02RX0JZ,02RX0KZ,02RX47Z,02RX48Z,02RX4JZ,02RX4KZ,03R007Z,03R00JZ,03R00KZ,03R047Z,03R04JZ,03R04KZ,03R107Z,03R10JZ,03R10KZ,03R147Z,03R14JZ,03R14KZ,03R207Z,03R20JZ,03R20KZ,03R247Z,03R24JZ,03R24KZ,03R307Z,03R30JZ,03R30KZ,03R347Z,03R34JZ,03R34KZ,03R407Z,03R40JZ,03R40KZ,03R447Z,03R44JZ,03R44KZ,05R007Z,05R00JZ,05R00KZ,05R047Z,05R04JZ,05R04KZ,05R107Z,05R10JZ,05R10KZ,05R147Z,05R14JZ,05R14KZ,05R307Z,05R30JZ,05R30KZ,05R347Z,05R34JZ,05R34KZ,05R407Z,05R40JZ,05R40KZ,05R447Z,05R44JZ,05R44KZ,05R507Z,05R50JZ,05R50KZ,05R547Z,05R54JZ,05R54KZ,05R607Z,05R60JZ,05R60KZ,05R647Z,05R64JZ,05R64KZ |
| Resection of aorta or other thoracic vessels with anastomosis | 38.34-38.35 | 02BW0ZZ,02BW4ZZ,02BX0ZZ,02BX4ZZ,04B00ZZ,04B04ZZ,02BP0ZZ,02BP4ZZ,02BQ0ZZ,02BQ4ZZ,02BR0ZZ,02BR4ZZ,02BS0ZZ,02BS4ZZ,02BT0ZZ,02BT4ZZ,02BV0ZZ,02BV4ZZ,03B00ZZ,03B04ZZ,03B10ZZ,03B14ZZ,03B20ZZ,03B24ZZ,03B30ZZ,03B34ZZ,03B40ZZ,03B44ZZ,05B00ZZ,05B04ZZ,05B10ZZ,05B14ZZ,05B30ZZ,05B34ZZ,05B40ZZ,05B44ZZ,05B50ZZ,05B54ZZ,05B60ZZ,05B64ZZ |
| Repair of blood vessel with synthetic patch graft or unspecified type of patch graft | 39.57-39.58 | 02UP0JZ,02UP3JZ,02UP4JZ,02UQ0JZ,02UQ3JZ,02UQ4JZ,02UR0JZ,02UR3JZ,02UR4JZ,02US0JZ,02US3JZ,02US4JZ,02UT0JZ,02UT3JZ,02UT4JZ,02UV0JZ,02UV3JZ,02UV4JZ,02UW0JZ,02UX0JZ,03U00JZ,03U03JZ,03U04JZ,03U10JZ,03U13JZ,03U14JZ,03U20JZ,03U23JZ,03U24JZ,03U30JZ,03U33JZ,03U34JZ,03U40JZ,03U43JZ,03U44JZ,03U50JZ,03U53JZ,03U54JZ,03U60JZ,03U63JZ,03U64JZ,03U70JZ,03U73JZ,03U74JZ,03U80JZ,03U83JZ,03U84JZ,03U90JZ,03U93JZ,03U94JZ,03UA0JZ,03UA3JZ,03UA4JZ,03UB0JZ,03UB3JZ,03UB4JZ,03UC0JZ,03UC3JZ,03UC4JZ,03UD0JZ,03UD3JZ,03UD4JZ,03UF0JZ,03UF3JZ,03UF4JZ,03UG0JZ,03UG3JZ,03UG4JZ,03UH0JZ,03UH3JZ,03UH4JZ,03UJ0JZ,03UJ3JZ,03UJ4JZ,03UK0JZ,03UK3JZ,03UK4JZ,03UL0JZ,03UL3JZ,03UL4JZ,03UM0JZ,03UM3JZ,03UM4JZ,03UN0JZ,03UN3JZ,03UN4JZ,03UP0JZ,03UP3JZ,03UP4JZ,03UQ0JZ,03UQ3JZ,03UQ4JZ,03UR0JZ,03UR3JZ,03UR4JZ,03US0JZ,03US3JZ,03US4JZ,03UT0JZ,03UT3JZ,03UT4JZ,03UU0JZ,03UU3JZ,03UU4JZ,03UV0JZ,03UV3JZ,03UV4JZ,03UY0JZ,03UY3JZ,03UY4JZ,04U00JZ,04U03JZ,04U04JZ,04U10JZ,04U13JZ,04U14JZ,04U20JZ,04U23JZ,04U24JZ,04U30JZ,04U33JZ,04U34JZ,04U40JZ,04U43JZ,04U44JZ,04U50JZ,04U53JZ,04U54JZ,04U60JZ,04U63JZ,04U64JZ,04U70JZ,04U73JZ,04U74JZ,04U80JZ,04U83JZ,04U84JZ,04U90JZ,04U93JZ,04U94JZ,04UA0JZ,04UA3JZ,04UA4JZ,04UB0JZ,04UB3JZ,04UB4JZ,04UC0JZ,04UC3JZ,04UC4JZ,04UD0JZ,04UD3JZ,04UD4JZ,04UE0JZ,04UE3JZ,04UE4JZ,04UF0JZ,04UF3JZ,04UF4JZ,04UH0JZ,04UH3JZ,04UH4JZ,04UJ0JZ,04UJ3JZ,04UJ4JZ,04UK0JZ,04UK3JZ,04UK4JZ,04UL0JZ,04UL3JZ,04UL4JZ,04UM0JZ,04UM3JZ,04UM4JZ,04UN0JZ,04UN3JZ,04UN4JZ,04UP0JZ,04UP3JZ,04UP4JZ,04UQ0JZ,04UQ3JZ,04UQ4JZ,04UR0JZ,04UR3JZ,04UR4JZ,04US0JZ,04US3JZ,04US4JZ,04UT0JZ,04UT3JZ,04UT4JZ,04UU0JZ,04UU3JZ,04UU4JZ,04UV0JZ,04UV3JZ,04UV4JZ,04UW0JZ,04UW3JZ,04UW4JZ,04UY0JZ,04UY3JZ,04UY4JZ,05U00JZ,05U03JZ,05U04JZ,05U10JZ,05U13JZ,05U14JZ,05U30JZ,05U33JZ,05U34JZ,05U40JZ,05U43JZ,05U44JZ,05U50JZ,05U53JZ,05U54JZ,05U60JZ,05U63JZ,05U64JZ,05U70JZ,05U73JZ,05U74JZ,05U80JZ,05U83JZ,05U84JZ,05U90JZ,05U93JZ,05U94JZ,05UA0JZ,05UA3JZ,05UA4JZ,05UB0JZ,05UB3JZ,05UB4JZ,05UC0JZ,05UC3JZ,05UC4JZ,05UD0JZ,05UD3JZ,05UD4JZ,05UF0JZ,05UF3JZ,05UF4JZ,05UG0JZ,05UG3JZ,05UG4JZ,05UH0JZ,05UH3JZ,05UH4JZ,05UL0JZ,05UL3JZ,05UL4JZ,05UM0JZ,05UM3JZ,05UM4JZ,05UN0JZ,05UN3JZ,05UN4JZ,05UP0JZ,05UP3JZ,05UP4JZ,05UQ0JZ,05UQ3JZ,05UQ4JZ,05UR0JZ,05UR3JZ,05UR4JZ,05US0JZ,05US3JZ,05US4JZ,05UT0JZ,05UT3JZ,05UT4JZ,05UV0JZ,05UV3JZ,05UV4JZ,05UY0JZ,05UY3JZ,05UY4JZ,06U00JZ,06U03JZ,06U04JZ,06U10JZ,06U13JZ,06U14JZ,06U20JZ,06U23JZ,06U24JZ,06U30JZ,06U33JZ,06U34JZ,06U40JZ,06U43JZ,06U44JZ,06U50JZ,06U53JZ,06U54JZ,06U60JZ,06U63JZ,06U64JZ,06U70JZ,06U73JZ,06U74JZ,06U80JZ,06U83JZ,06U84JZ,06U90JZ,06U93JZ,06U94JZ,06UB0JZ,06UB3JZ,06UB4JZ,06UC0JZ,06UC3JZ,06UC4JZ,06UD0JZ,06UD3JZ,06UD4JZ,06UF0JZ,06UF3JZ,06UF4JZ,06UG0JZ,06UG3JZ,06UG4JZ,06UH0JZ,06UH3JZ,06UH4JZ,06UJ0JZ,06UJ3JZ,06UJ4JZ,06UM0JZ,06UM3JZ,06UM4JZ,06UN0JZ,06UN3JZ,06UN4JZ,06UP0JZ,06UP3JZ,06UP4JZ,06UQ0JZ,06UQ3JZ,06UQ4JZ,06UR0JZ,06UR3JZ,06UR4JZ,06US0JZ,06US3JZ,06US4JZ,06UT0JZ,06UT3JZ,06UT4JZ,06UV0JZ,06UV3JZ,06UV4JZ,06UY0JZ,06UY3JZ,06UY4JZ,02UP07Z,02UP08Z,02UP0JZ,02UP0KZ,02UP37Z,02UP3JZ,02UP3KZ,02UP47Z,02UP4JZ,02UP4KZ,02UQ07Z,02UQ08Z,02UQ0JZ,02UQ0KZ,02UQ37Z,02UQ38Z,02UQ3JZ,02UQ3KZ,02UQ47Z,02UQ48Z,02UQ4JZ,02UQ4KZ,02UR07Z,02UR08Z,02UR0JZ,02UR0KZ,02UR37Z,02UR38Z,02UR3JZ,02UR3KZ,02UR47Z,02UR48Z,02UR4JZ,02UR4KZ,02US07Z,02US08Z,02US0JZ,02US0KZ,02US37Z,02US38Z,02US3JZ,02US3KZ,02US47Z,02US48Z,02US4JZ,02US4KZ,02UT07Z,02UT08Z,02UT0JZ,02UT0KZ,02UT37Z,02UT38Z,02UT3JZ,02UT3KZ,02UT47Z,02UT48Z,02UT4JZ,02UT4KZ,02UV07Z,02UV08Z,02UV0JZ,02UV0KZ,02UV37Z,02UV38Z,02UV3JZ,02UV3KZ,02UV47Z,02UV48Z,02UV4JZ,02UV4KZ,02UW07Z,02UW08Z,02UW0JZ,02UW0KZ,02UW37Z,02UW38Z,02UW3JZ,02UW3KZ,02UW47Z,02UW48Z,02UW4JZ,02UW4KZ,02UX07Z,02UX08Z,02UX0JZ,02UX0KZ,02UX37Z,02UX38Z,02UX3JZ,02UX3KZ,02UX47Z,02UX48Z,02UX4JZ,02UX4KZ,03U00KZ,03U03KZ,03U04KZ,03U10KZ,03U13KZ,03U14KZ,03U20KZ,03U23KZ,03U24KZ,03U30KZ,03U33KZ,03U34KZ,03U40KZ,03U43KZ,03U44KZ,03U50KZ,03U53KZ,03U54KZ,03U60KZ,03U63KZ,03U64KZ,03U70KZ,03U73KZ,03U74KZ,03U80KZ,03U83KZ,03U84KZ,03U90KZ,03U93KZ,03U94KZ,03UA0KZ,03UA3KZ,03UA4KZ,03UB0KZ,03UB3KZ,03UB4KZ,03UC0KZ,03UC3KZ,03UC4KZ,03UD0KZ,03UD3KZ,03UD4KZ,03UF0KZ,03UF3KZ,03UF4KZ,03UG0KZ,03UG3KZ,03UG4KZ,03UH0KZ,03UH3KZ,03UH4KZ,03UJ0KZ,03UJ3KZ,03UJ4KZ,03UK0KZ,03UK3KZ,03UK4KZ,03UL0KZ,03UL3KZ,03UL4KZ,03UM0KZ,03UM3KZ,03UM4KZ,03UN0KZ,03UN3KZ,03UN4KZ,03UP0KZ,03UP3KZ,03UP4KZ,03UQ0KZ,03UQ3KZ,03UQ4KZ,03UR0KZ,03UR3KZ,03UR4KZ,03US0KZ,03US3KZ,03US4KZ,03UT0KZ,03UT3KZ,03UT4KZ,03UU0KZ,03UU3KZ,03UU4KZ,03UV0KZ,03UV3KZ,03UV4KZ,03UY0KZ,03UY3KZ,03UY4KZ,04U00KZ,04U03KZ,04U04KZ,04U10KZ,04U13KZ,04U14KZ,04U20KZ,04U23KZ,04U24KZ,04U30KZ,04U33KZ,04U34KZ,04U40KZ,04U43KZ,04U44KZ,04U50KZ,04U53KZ,04U54KZ,04U60KZ,04U63KZ,04U64KZ,04U70KZ,04U73KZ,04U74KZ,04U80KZ,04U83KZ,04U84KZ,04U90KZ,04U93KZ,04U94KZ,04UA0KZ,04UA3KZ,04UA4KZ,04UB0KZ,04UB3KZ,04UB4KZ,04UC0KZ,04UC3KZ,04UC4KZ,04UD0KZ,04UD3KZ,04UD4KZ,04UE0KZ,04UE3KZ,04UE4KZ,04UF0KZ,04UF3KZ,04UF4KZ,04UH0KZ,04UH3KZ,04UH4KZ,04UJ0KZ,04UJ3KZ,04UJ4KZ,04UK0KZ,04UK3KZ,04UK4KZ,04UL0KZ,04UL3KZ,04UL4KZ,04UM0KZ,04UM3KZ,04UM4KZ,04UN0KZ,04UN3KZ,04UN4KZ,04UP0KZ,04UP3KZ,04UP4KZ,04UQ0KZ,04UQ3KZ,04UQ4KZ,04UR0KZ,04UR3KZ,04UR4KZ,04US0KZ,04US3KZ,04US4KZ,04UT0KZ,04UT3KZ,04UT4KZ,04UU0KZ,04UU3KZ,04UU4KZ,04UV0KZ,04UV3KZ,04UV4KZ,04UW0KZ,04UW3KZ,04UW4KZ,04UY0KZ,04UY3KZ,04UY4KZ,05U00KZ,05U03KZ,05U04KZ,05U10KZ,05U13KZ,05U14KZ,05U30KZ,05U33KZ,05U34KZ,05U40KZ,05U43KZ,05U44KZ,05U50KZ,05U53KZ,05U54KZ,05U60KZ,05U63KZ,05U64KZ,05U70KZ,05U73KZ,05U74KZ,05U80KZ,05U83KZ,05U84KZ,05U90KZ,05U93KZ,05U94KZ,05UA0KZ,05UA3KZ,05UA4KZ,05UB0KZ,05UB3KZ,05UB4KZ,05UC0KZ,05UC3KZ,05UC4KZ,05UD0KZ,05UD3KZ,05UD4KZ,05UF0KZ,05UF3KZ,05UF4KZ,05UG0KZ,05UG3KZ,05UG4KZ,05UH0KZ,05UH3KZ,05UH4KZ,05UL0KZ,05UL3KZ,05UL4KZ,05UM0KZ,05UM3KZ,05UM4KZ,05UN0KZ,05UN3KZ,05UN4KZ,05UP0KZ,05UP3KZ,05UP4KZ,05UQ0KZ,05UQ3KZ,05UQ4KZ,05UR0KZ,05UR3KZ,05UR4KZ,05US0KZ,05US3KZ,05US4KZ,05UT0KZ,05UT3KZ,05UT4KZ,05UV0KZ,05UV3KZ,05UV4KZ,05UY0KZ,05UY3KZ,05UY4KZ,06U00KZ,06U03KZ,06U04KZ,06U10KZ,06U13KZ,06U14KZ,06U20KZ,06U23KZ,06U24KZ,06U30KZ,06U33KZ,06U34KZ,06U40KZ,06U43KZ,06U44KZ,06U50KZ,06U53KZ,06U54KZ,06U60KZ,06U63KZ,06U64KZ,06U70KZ,06U73KZ,06U74KZ,06U80KZ,06U83KZ,06U84KZ,06U90KZ,06U93KZ,06U94KZ,06UB0KZ,06UB3KZ,06UB4KZ,06UC0KZ,06UC3KZ,06UC4KZ,06UD0KZ,06UD3KZ,06UD4KZ,06UF0KZ,06UF3KZ,06UF4KZ,06UG0KZ,06UG3KZ,06UG4KZ,06UH0KZ,06UH3KZ,06UH4KZ,06UJ0KZ,06UJ3KZ,06UJ4KZ,06UM0KZ,06UM3KZ,06UM4KZ,06UN0KZ,06UN3KZ,06UN4KZ,06UP0KZ,06UP3KZ,06UP4KZ,06UQ0KZ,06UQ3KZ,06UQ4KZ,06UR0KZ,06UR3KZ,06UR4KZ,06US0KZ,06US3KZ,06US4KZ,06UT0KZ,06UT3KZ,06UT4KZ,06UV0KZ,06UV3KZ,06UV4KZ,06UY0KZ,06UY3KZ,06UY4KZ |
| **Cardiac surgery** |  |  |
| Cardioplegia | 39.63 | 3E080GC,3E083GC |
| Valve repair | 35.00-35.99 | 02NF3ZZ,02NF4ZZ,02NG3ZZ,02NG4ZZ,02NH3ZZ,02NH4ZZ,02NJ3ZZ,02NJ4ZZ,02NF3ZZ,02NF4ZZ,02NG3ZZ,02NG4ZZ,02NH3ZZ,02NH4ZZ,02NJ3ZZ,02NJ4ZZ,02RF37Z,02RF38Z,02RF3JZ,02RF3KZ,X2RF332,02RF37H,02RF38H,02RF3JH,02RF3KH,02RH37Z,02RH38Z,02RH3JZ,02RH3KZ,02RH37H,02RH38H,02RH3JH,02RH3KH,02RF37Z,02RF38Z,02RF3JZ,02RF3KZ,02RG37H,02RG37Z,02RG38H,02RG38Z,02RG3JH,02RG3JZ,02RG3KH,02RG3KZ,02RH37Z,02RH38Z,02RH3JZ,02RH3KZ,02QF0ZZ,02QG0ZZ,02QH0ZZ,02QJ0ZZ,027F04Z,027F0DZ,027F0ZZ,02NF0ZZ,02QF0ZZ,027G04Z,027G0DZ,027G0ZZ,02NG0ZZ,02QG0ZZ,027H04Z,027H0DZ,027H0ZZ,02NH0ZZ,02QH0ZZ,027J04Z,027J0DZ,027J0ZZ,02NJ0ZZ,02QJ0ZZ,02RF07Z,02RF08Z,02RF0JZ,02RF0KZ,02RF47Z,02RF48Z,02RF4JZ,02RF4KZ,02RG07Z,02RG08Z,02RG0JZ,02RG0KZ,02RG47Z,02RG48Z,02RG4JZ,02RG4KZ,02RH07Z,02RH08Z,02RH0JZ,02RH0KZ,02RH47Z,02RH48Z,02RH4JZ,02RH4KZ,02RJ07Z,02RJ08Z,02RJ0JZ,02RJ0KZ,02RJ47Z,02RJ48Z,02RJ4JZ,02RJ4KZ,02RF07Z,02RF08Z,02RF0KZ,02RF47Z,02RF48Z,02RF4KZ,X2RF032,X2RF432,02RF0JZ,02RF4JZ,02RG07Z,02RG08Z,02RG0KZ,02RG37Z,02RG38Z,02RG3KZ,02RG47Z,02RG48Z,02RG4KZ,02RG0JZ,02RG3JZ,02RG4JZ,02RH07Z,02RH08Z,02RH0KZ,02RH47Z,02RH48Z,02RH4KZ,02RH0JZ,02RH4JZ,02RJ07Z,02RJ08Z,02RJ0KZ,02RJ47Z,02RJ48Z,02RJ4KZ,02RJ0JZ,02RJ4JZ,028D0ZZ,028D3ZZ,028D4ZZ,02QD0ZZ,02QD3ZZ,02QD4ZZ,02890ZZ,02893ZZ,02894ZZ,02Q90ZZ,02Q93ZZ,02Q94ZZ,02QF0ZZ,02QF3ZZ,02QF4ZZ,02QG0ZZ,02QG3ZZ,02QG4ZZ,02QH0ZZ,02QH3ZZ,02QH4ZZ,02QJ0ZZ,02QJ3ZZ,02QJ4ZZ,02BK0ZZ,02BK3ZZ,02BK4ZZ,02NK0ZZ,02NK3ZZ,02NK4ZZ,02NL0ZZ,02NL3ZZ,02NL4ZZ,02QF0ZZ,02QF3ZZ,02QF4ZZ,02QA0ZZ,02QA3ZZ,02QA4ZZ,02B50ZZ,02B53ZZ,02B54ZZ,02RM0JZ,02RM4JZ,02U50JZ,02U53JZ,02U54JZ,02UM0JZ,02U50JZ,02U53JZ,02U54JZ,02RM0JZ,02UM0JZ,02UM3JZ,02UM4JZ,024G0J2,024J0J2,02QH4ZZ,02QJ4ZZ,02QJ3ZZ,02QJ0ZZ,02QH3ZZ,02QH0ZZ,02QG4ZZ,02QG3ZZ,02QG0ZZ,02QF4ZZ,02QF3ZZ,02QF0ZZ,02U50JZ,02U53JZ,02U54JZ,02UM0JZ,02UM3JZ,02UM4JZ,02RM4JZ,02RM07Z,02RM0KZ,02RM47Z,02RM4KZ,02U507Z,02U508Z,02U50KZ,02U537Z,02U538Z,02U53KZ,02U547Z,02U548Z,02U54KZ,02UM07Z,02UM0KZ,02UM37Z,02UM3KZ,02UM47Z,02UM4KZ,02U507Z,02U508Z,02U50KZ,02U537Z,02U538Z,02U53KZ,02U547Z,02U548Z,02U54KZ,02RM07Z,02RM0KZ,02RM47Z,02RM4KZ,02UM07Z,02UM0KZ,02UM38Z,02UM48Z,024G072,024G082,024G0K2,024J072,024J082,024J0K2,02RK07Z,02RK0KZ,02RK47Z,02RK4KZ,02RL07Z,02RL0KZ,02RL47Z,02RL4KZ,02U607Z,02U608Z,02U707Z,02U708Z,02U70KZ,02U737Z,02U738Z,02U73KZ,02U747Z,02U748Z,02U74KZ,02UK0KZ,02UK3KZ,02UK4KZ,02UL0KZ,02UL3KZ,02UL4KZ,02Q50ZZ,02Q53ZZ,02Q54ZZ,02QM0ZZ,02QM3ZZ,02QM4ZZ,02Q50ZZ,02Q53ZZ,02Q54ZZ,02QM0ZZ,02QM3ZZ,02QM4ZZ,02QB0ZZ,02QB3ZZ,02QB4ZZ,02QC0ZZ,02QC3ZZ,02QC4ZZ,02RM0JZ,02RP0JZ,02RQ0JZ,02RR0JZ,02BK0ZZ,02NH0ZZ,02170ZR,02170ZP,02170ZQ,02U70JZ,02LS0ZZ,02LT0ZZ,02RM0JZ,024F07J,024F08J,024F0JJ,024F0KJ,02RM0JZ,02LR0ZT,02VR0ZT,02RQ07Z,02RQ0JZ,02RR07Z,02RR0JZ,02S00ZZ,02S10ZZ,02SP0ZZ,02SW0ZZ,02SX0ZZ,02U50JZ,021K4AQ,021K4ZR,021K4ZQ,021K4ZP,021K4AR,021K4JP,021K4JQ,021K4KR,021K4JR,021K08P,021K4KP,021K4KQ,021K49R,021K08Q,021K08R,021K09P,021K09Q,021K09R,021K0AP,021K0AQ,021K0AR,021K0JP,021K0JQ,021K0JR,021K0KQ,021K4AP,021K49Q,021K49P,021K48R,021K48Q,021K48P,021K0ZR,021K0ZQ,021K0ZP,021K0KR,021K0KP,02LH0CZ,02LH0DZ,02LH0ZZ,02LH3CZ,02LH3DZ,02LH3ZZ,02LH4CZ,02LH4DZ,02LH4ZZ,021L0ZW,021L4ZW,021608P,021608Q,021608R,021609P,021609Q,021609R,02160AP,02160AQ,02160AR,02160JP,02160JQ,02160JR,02160KP,02160KQ,02160KR,02160ZP,02160ZQ,02160ZR,021648P,021648Q,021648R,021649P,021649Q,021649R,02164AP,02164AQ,02164AR,02164JP,02164JQ,02164JR,02164KP,02164KQ,02164KR,02164ZP,02164ZQ,02164ZR,02W50JZ,02W54JZ,02WF07Z,02WF08Z,02WF0JZ,02WF0KZ,02WF47Z,02WF48Z,02WF4JZ,02WF4KZ,02WG07Z,02WG08Z,02WG0JZ,02WG0KZ,02WG47Z,02WG48Z,02WG4JZ,02WG4KZ,02WH07Z,02WH08Z,02WH0JZ,02WH0KZ,02WH47Z,02WH48Z,02WH4JZ,02WH4KZ,02WJ07Z,02WJ08Z,02WJ0JZ,02WJ0KZ,02WJ47Z,02WJ48Z,02WJ4JZ,02WJ4KZ,02WM0JZ,02WM4JZ,027F3ZZ,027F4ZZ,027G3ZZ,027G4ZZ,027H3ZZ,027H4ZZ,027J3ZZ,027J4ZZ,02UG3JZ,02Q50ZZ,02Q53ZZ,02Q54ZZ,02QM0ZZ,02QM3ZZ,02QM4ZZ,02QF0ZJ,02QF0ZZ,02QF3ZJ,02QF4ZJ,02QG0ZE,02QG0ZZ,02QG3ZE,02QG4ZE,02QH0ZZ,02QJ0ZG,02QJ0ZZ,02QJ3ZG,02QJ4ZG |
| Operations on vessels of the heart | 36.00-36.99, 37.0, 37.20-37.29, 37.31-37.90, 37.93-37.99 | 0W9D30Z,0W9D3ZX,0W9D3ZZ,0W9D40Z,0W9D4ZX,0W9D4ZZ,4A02X4Z,4A02XFZ,4A020N6,4A023N6,4A020N7,4A023N7,4A020N8,4A023N8,02BN0ZX,02BN3ZX,02BN4ZX,02B40ZX,02B43ZX,02B44ZX,02B50ZX,02B53ZX,02B54ZX,02B60ZX,02B63ZX,02B64ZX,02B70ZX,02B73ZX,02B74ZX,02B80ZX,02B83ZX,02B84ZX,02B90ZX,02B93ZX,02B94ZX,02BD0ZX,02BD3ZX,02BD4ZX,02BF0ZX,02BF3ZX,02BF4ZX,02BG0ZX,02BG3ZX,02BG4ZX,02BH0ZX,02BH3ZX,02BH4ZX,02BJ0ZX,02BJ3ZX,02BJ4ZX,02BK0ZX,02BK3ZX,02BK4ZX,02BL0ZX,02BL3ZX,02BL4ZX,02BM0ZX,02BM3ZX,02BM4ZX,4A023FZ,3E053KZ,3E063KZ,02K80ZZ,02K83ZZ,02K84ZZ,B244YZZ,B244ZZZ,B245YZZ,B245ZZZ,B246YZZ,B246ZZZ,B24DYZZ,B24DZZZ,02JA3ZZ,0WJD0ZZ,0WJD3ZZ,02BN0ZZ,02BN3ZZ,02BN4ZZ,02TN0ZZ,02TN3ZZ,02TN4ZZ,02B60ZZ,02B63ZZ,02B64ZZ,02B70ZZ,02B73ZZ,02B74ZZ,02BK0ZZ,02BK3ZZ,02BK4ZZ,02BL0ZZ,02BL3ZZ,02BL4ZZ,02560ZZ,02570ZZ,025K0ZZ,025L0ZZ,02B60ZZ,02B70ZZ,02BK0ZZ,02BL0ZZ,02T80ZZ,02563ZZ,02573ZZ,025K3ZZ,025L3ZZ,02B63ZZ,02B73ZZ,02BK3ZZ,02BL3ZZ,02BK0ZZ,02BK3ZZ,02BK4ZZ,02BL0ZZ,02BL3ZZ,02BL4ZZ,02570ZK,02573ZK,02574ZK,02B70ZK,02B73ZK,02B74ZK,02L70ZK,02L73ZK,02L74ZK,02564ZZ,02574ZZ,025K4ZZ,025L4ZZ,02B64ZZ,02B74ZZ,02BK4ZZ,02BL4ZZ,02UA0JZ,02UA3JZ,02UA4JZ,02QA0ZZ,02QA3ZZ,02QA4ZZ,02QN0ZZ,02QN3ZZ,02QN4ZZ,02YA0Z0,02YA0Z1,02YA0Z2,02RK0JZ,02RL0JZ,02RK0JZ,02RL0JZ,02WA0JZ,02PA0JZ,5A02110,5A02210,02WA0QZ,02WA0RZ,02WA3QZ,02WA3RZ,02WA4QZ,02WA4RZ,02PA0QZ,02PA0RZ,02PA3QZ,02PA3RZ,02PA4QZ,02PA4RZ,02HA0QZ,02HA3QZ,02HA4QZ,02QA0ZZ,02QA3ZZ,02QA4ZZ,5A02116,5A0211D,5A02216,5A0221D,02HA3RZ,5A02216,02H40JZ,02H40MZ,02H44JZ,02H44MZ,02H60JZ,02H60MZ,02H63JZ,02H64JZ,02H64MZ,02H70JZ,02H70MZ,02H73JZ,02H74JZ,02H74MZ,02HK0JZ,02HK0MZ,02HK3JZ,02HK4JZ,02HK4MZ,02HL0JZ,02HL0MZ,02HL3JZ,02HL4JZ,02HL4MZ,02HK3JZ,02HK3MZ,02HL3JZ,02HL3MZ,02H63JZ,02H73JZ,02HN0JZ,02HN0MZ,02HN3JZ,02HN3MZ,02HN4JZ,02HN4MZ,02WA0MZ,02WA3MZ,02WA4MZ,02H63JZ,02H63MZ,02H73JZ,02H73MZ,02HK3JZ,02HL3JZ,02PA0MZ,02PA3MZ,02PA4MZ,02PAXMZ,5A1213Z,5A1223Z,0JH602Z,0JH632Z,0JWT02Z,0JWT0PZ,0JWT32Z,0JWT3PZ,0JH60PZ,0JH63PZ,0JH80PZ,0JH83PZ,0JH604Z,0JH634Z,0JH804Z,0JH834Z,0JH605Z,0JH635Z,0JH805Z,0JH835Z,0JH606Z,0JH636Z,0JH806Z,0JH836Z,0JPT0PZ,0JPT3PZ,0JWT0PZ,0JWT3PZ,02L70CK,02L70DK,02L73CK,02L73DK,02L74CK,02L74DK,02QA0ZZ,3E070GC,3E073GC,3E080GC,3E083GC,0JH608Z,0JH638Z,0JH838Z,0JH808Z,02H74KZ,02HL4KZ,02HL3KZ,02HL0KZ,02HK4KZ,02HK3KZ,02HK0KZ,02H73KZ,02H70KZ,02H64KZ,02H63KZ,02H60KZ,0JH808Z,0JH638Z,0JH608Z,0JH838Z,0JPT3PZ,0JPT0PZ,02PA0MZ,02PA3MZ,02PA4MZ,02PAXMZ,02HL3KZ,02HL4KZ,02HL0KZ,02HK4KZ,02HK3KZ,02HK0KZ,02H74KZ,02H73KZ,02H70KZ,02H64KZ,02H63KZ,02H60KZ,02H63KZ,02H73KZ,02HK3KZ,02HL3KZ,02HN0KZ,02HN4KZ,0JH608Z,0JH638Z,0JH808Z,0JH838Z,02H44KZ,02HN4KZ,02HN3KZ,02HN0KZ,02HL4KZ,02HL3KZ,02HL0KZ,02HK4KZ,02HK3KZ,02HK0KZ,02H74KZ,02H73KZ,02H70KZ,02H64KZ,02H63KZ,02H60KZ,02H40KZ,02PA0MZ,02PA3MZ,02PA4MZ,02PAXMZ,0JH608Z,0JH638Z,0JH808Z,0JH838Z,0JPT0PZ,0JPT3PZ,02QA0ZZ,02QA3ZZ,02QA4ZZ,0JPT0PZ,0JPT3PZ,0JWT0PZ,0JWT3PZ,02700ZZ,02710ZZ,02720ZZ,02730ZZ,02C00ZZ,02C10ZZ,02C20ZZ,02C30ZZ,3E07017,3E070PZ,3E07317,3E073PZ,02C03ZZ,02C04ZZ,02C13ZZ,02C14ZZ,02C23ZZ,02C24ZZ,02C33ZZ,02C34ZZ,0210093,02100A3,02100J3,02100K3,02100Z3,0210493,02104A3,02104J3,02104K3,02104Z3,021008W,021009W,02100AW,02100JW,02100KW,021048W,021049W,02104AW,02104JW,02104KW,021108W,021109W,02110AW,02110JW,02110KW,021148W,021149W,02114AW,02114JW,02114KW,021208W,021209W,02120AW,02120JW,02120KW,021248W,021249W,02124AW,02124JW,02124KW,021308W,021309W,02130AW,02130JW,02130KW,021348W,021349W,02134AW,02134JW,02134KW,021008F,021009F,02100AF,02100JF,02100KF,02100ZF,021048F,021049F,02104AF,02104JF,02104KF,02104ZF,0210083,0210093,02100A3,02100J3,02100K3,02100Z3,0210483,0210493,02104A3,02104J3,02104K3,02104Z3,021K0Z5,021L0Z5,021K4Z5,021L4Z5,02QA4ZZ,02QB4ZZ,02QC4ZZ,02QA3ZZ,02QB3ZZ,02QC3ZZ,0210344,02103D4,0210444,02104D4,0211344,02113D4,0211444,02114D4,0212344,02123D4,0212444,02124D4,0213344,02133D4,0213444,02134D4,02QA0ZZ,02QB0ZZ,02QC0ZZ,02Q00ZZ,02Q03ZZ,02Q04ZZ,02Q40ZZ,02Q43ZZ,02Q44ZZ,02Q00ZZ,02Q03ZZ,02Q04ZZ,02Q40ZZ,02Q43ZZ,02Q44ZZ,02HA0RS,02HA3RS,02HA4RS,5A02116,5A02216,02HA0RZ,02HA3RZ,02HA4RZ,5A02116,5A02216,02HA0RZ,02HA4RZ,5A02116,5A02216,02HK3JZ,02HL3JZ,02H63JZ,02H73JZ,02HN0JZ,02HN0MZ,02HN3JZ,02HN3MZ,02HN4JZ,02HN4MZ,02PA0MZ,02PA3MZ,02PA4MZ,02PAXMZ,02H63JZ,02H73JZ,02HK3JZ,02HK3MZ,02HL3JZ,02HL3MZ,02PA0MZ,02PA3MZ,02PA4MZ,02PAXMZ,0JH60PZ,0JH63PZ,0JH80PZ,0JH83PZ,0JPT0PZ,0JPT3PZ,0JH604Z,0JH634Z,0JH804Z,0JH834Z,0JPT0PZ,0JPT3PZ,0JH605Z,0JH635Z,0JH805Z,0JH835Z,0JPT0PZ,0JPT3PZ,0JH606Z,0JH636Z,0JH806Z,0JH836Z,0JPT0PZ,0JPT3PZ |
| TEVAR | 39.73 | 02UW3JZ,02UW4JZ,02UX3JZ,02UX4JZ,02VW0DZ,02VW3DZ,02VW4DZ,02VX0DZ,02VX3DZ,02VX4DZ |
| **Comorbidity** | **ICD-9** | **ICD-10** |
| Chronic liver disease | 571.0-571.6, 571.8, 571.9, 571.40-571.42, 571.49 | K70.0, K70.10, K70.30, K70.9, K73.9, K73.0, K75.4, K73.2, K73.8, K74.0, K74.60, K74.69, K74.3, K74.4, K74.5, K76.0, K76.89 |
| Type 2 diabetes | 250.00, 250.02, 250.10, 250.12, 250.20, 250.22, 250.30, 250.32, 250.40, 250.42, 250.50, 250.52, 250.60, 250.62, 250.70, 250.72, 250.80, 250.82, 250.90, 250.92 | E11.9, E11.65, E11.69, E13.10, E11.00, E11.01, E11.641, E11.29, E11.21, E11.311, E11.319, E11.36, E11.39, E11.40, E11.51, E11.618, E11.620, E11.621, E11.622, E11.628, E11.630, E11.638, E11.649, E11.8 |
| Hypertension | 401.1, 401.9, 642.00-642.04, 401.0, 402.00, 402.10, 402.90, 403.00, 403.10, 403.90, 404.00, 404.10, 404.90, 405.01, 405.09, 405.11, 405.19, 405.91, 405.99, 642.10-642.24, 642.70-642.94 | I10, I16.9, O10.019, O10.919, O10.011, O10.012, O10.013, O10.02, O10.911, O10.912, O10.913, O10.92, O10.03, O10.93, I11.9, I12.9, I13.10, I15.0, I15.8, O10.419, O10.411, O10.412, O10.413, O10.42, O10.43, O10.119, O10.219, O10.319, O11.9, O10.111, O10.112, O10.113, O10.12, O10.211, O10.212, O10.213, O10.22, O10.311, O10.312, O10.313, O10.32, O11.1, O11.2, O11.3, O10.13, O10.23, O10.33, O11.4, O11.5, O16.9, O16.1, O16.2, O16.3, O16.4, O16.5 |
| Dyslipidemia | 272.0, 272.1, 272.2, 272.3, 272.4 | E78.00, E78.01, E78.1, E78.3, E78.4, E78.5 |
| Chronic kidney disease | 403.01, 403.11, 403.91, 404.02, 404.03, 404.12, 404.13, 404.92, 404.93, 582.x, 583.0-583.7, 585.x, 586.x, 588.0, V42.0, V45.1, V56.x | I12.0, I13.1, N03.2-N03.7, N05.2-N05.7, N18.x, N19.x, N25.0, Z49.0-Z49.2, Z94.0, Z99.2 |
| Coronary artery disease (CAD) | 414.00-414.7 | I25.10, I25.810, I25.811, I25.812 |
| Heart failure | 398.91, 402.01, 402.11, 402.91, 404.01, 404.11, 404.91, 428.20, 428.21, 428.22, 428.23, 428.0, 428.1, 428.30, 428.31, 428.32, 428.33, 428.40, 428.41, 428.42, 428.43, 428.9 | I09.81, I11.0, I13.0, I50.9, I50.1, I50.20, I50.21, I50.22, I50.23, I50.30, I50.31, I50.32, I50.33, I50.40, I50.41, I50.42, I50.43, I50.9 |
| Stroke | 997.02, 431, 432.1, 434.91, 434.11, 434.01 | I61.9, I97.811, I97.812, I62.00, I63.50, I63.30, I63.40 |
| COPD | 416.8, 416.9, 490.x-505.x, 506.4, 508.1, 508.8 | I27.8, I27.9, J40.x-J47.x, J60.x-J67.x, J68.4, J70.1, J70.3 |
| Coagulopathy | 286.0-286.9, 287.1, 287.3-287.5, 289.81-289.82, 286.52, 286.53, 286.59, 287.30, 287.31, 287.32, 287.33, 287.39, 287.41, 287.49 | D66, D67, D68.1, D68.2, D68.0, D68.311, D68.312, D68.318, D65, D68.32, D68.4, D68.8, D68.9, D69.1, D69.49, D69.3, D69.41, D69.42, D69.3, D69.49, D69.51, D69.59, D69.6, D68.51, D68.52, D68.59, D68.61, D68.62, D68.69 |
| Anemia | 280.1, 281.8, 281.9, 285.21, 285.22, 285.29, 285.9 | D50.8, D50.1, D50.9, D63.1, D63.0, D63.8, D64.9 |
| Peripheral neurologic disorders | 330.0-331.9, 332.0, 333.4, 333.5, 334.0-335.9, 340, 341.1-341.9, 345.00-345.11, 345.2-345.3, 345.40-345.91, 348.1, 348.3-348.39, 780.3, 784.3 | E75.23, E75.25, E75.29, E75.02, E75.19, E75.4, G93.89, G93.9, F84.2,G31.81, G31.82, G31.9, G30.9, G31.01, G31.09, G31.1, G91.0, G91.1,G91.2, G31.85, G94, G93.7, G31.83, G31.84, G31.89, G20, G10, G25.4, G25.5, G11.1, G11.4, G11.0, G11.2, G32.81, G11.3, G11.8, G11.9, G12.0, G12.9, G12.1, G12.8, G12.21, G12.22, G12.29, G35, G37.0, G37.5, G37.3, G37.1, G37.2, G37.8, G37.9, G40.A01, G40.A09, G40.A11, G40.A19, G40.309, G40.409, G40.401, G40.311, G40.411, G40.419, G40.301, G40.201, G40.209, G40.101, G40.109, G40.821, G40.821, G40.822, G40.823, G40.824, G40.111, G40.119, G40.501, G40.509, G40.802, G40.804, G40.901, G40.909, G40.911, G40.919, G93.1, G93.40, G93.41, G93.49, I67.83, R56.00, R56.01, R56.1, R56.9,R47.01 |
| Atrial fibrillation | 427.3x | I48.91, I48.92 |
| Obesity | 278.0, 278.00, 278.01 | E66.9, E66.01, E66.3, E66.2 |
| Peripheral vascular disorders | 440.0-440.9, 441.00-441.9, 442.0-442.9, 443.1-443.9, 447.1, 557.1, 557.9, V43.4 | I70.0, I70.1, |
| Marfan syndrome | 759.82 | Q87.40 |
|  |  |  |
| **Complication** | **ICD-9** | **ICD-10** |
| **Respiratory** |  |  |
| Hypoxia | 799.02 | R09.02 |
| Pneumonia | 481, 482.0, 482.1, 482.2, 482.30, 482.31, 482.32, 482.39, 482.40, 482.41, 482.42, 482.49, 482.81, 482.82, 482.83, 482.89, 482.9, 483.0, 485, 486, 507.0, 997.31, 997.32 | J13, J18.1, J15.0, J15.1, J14, J15.4, J15.4, J15.3, J15.4, J15.20, J15.211, J15.212, J15.29, J15.8, J15.5, J15.6, J15.8, J15.9, J15.7, J18.0, J18.9, J69.0, J95.851, J95.89 |
| Prolonged ventilation (>96h)a | 96.72 | 5A1955Z |
| Perioperative reintubation | 96.04, 96.05 | 0BH17EZ,0BH18EZ,0B717DZ,0B718DZ,0BH07DZ,0WHQ7YZ |
| Acute respiratory distress syndrome | 518.82 | J80 |
| Respiratory failure | 518.5, 518.81, 518.83, 518.84 | J95.821, J96.00, J95.1, J95.2, J95.3, J96.00, J96.90, J96.20, J96.10 |
| **Cardiac** |  |  |
| Cardiac arrest | 427.5 | I46.9 |
| Prioperative arrhythmia | 427.1, 427.41, 427.42, 427.31, 427.0, 427.89, 427.32 | I47.2, I49.01, I49.02, I48.91, I47.1, I49.8, R00.1, I48.92 |
| Heart conduction disturbance | 997.1 | I97.710, I97.790, I97.88, I97.89 |
| Perioperative myocardial infarction | 410.1-410.6, 410.81, 410.91, 410.71 | I21.09, I21.19, I21.11, I21.29, I21.4, I21.3 |
| Mitral/aortic stenosis/insufficiency | 396.0, 396.1, 396.2, 396.3 | I08.0 |
| Tricuspid insufficiency | 397.0 | I07.1, I07.2, I07.8 |
| Pericardial effusion | 423.0 | I31.2 |
| Cardiac tamponade | 423.3 | I31.4 |
| **Renal** |  |  |
| Peritoneal dialysis | 54.98 | 3E1M39Z |
| Hemodialysis | 39.95 | 5A1D00Z, 5A1D60Z, 5A1D70Z, 5A1D80Z, 5A1D90Z |
| Renal failure (requiring renal replacement therapy) | V42.0, V45.1, V56 | Z94.0, Z99.2, Z91.15, Z49.31, Z49.32 |
| **Permanent neurological deficits** |  |  |
| Stroke | 433.01, 433.10, 433.11, 433.21, 433.31, 433.81, 433.91, 434.01, 434.11, 434.91, 436, 430, 431, 432.9 | I63.22, I65.29, I63.139, I63.239, I63.019, I63.119, I63.219, I63.59, I63.20, I63.30, I63.40, I63.50, I67.89, I60.9, I61.9, I62.9 |
|  |  |  |
| Hemiplegia or paraplegia | 344.1, 342.x | G82.20, G81.00, G81.01, G81.02, G81.03, G81.04, G81.10, G81.11, G81.12, G81.13, G81.14, G81.90, G81.91, G81.92, G81.93, G81.94 |
| Spinal cord complications | 952.0x, 952.1x | S14.101A, S14.102A, S14.103A, S14.104A, S14.105A, S14.106A, S14.107A, S14.108A, S14.111A, S14.112A, S14.113A, S14.114A, S14.115A, S14.116A, S14.117A, S14.118A, S14.131A, S14.132A, S14.133A, S14.134A, S14.135A, S14.136A, S14.137A, S14.138A, S14.121A, S14.122A, S14.123A, S14.124A, S14.125A, S14.126A, S14.127A, S14.128A, S14.151A, S14.152A, S14.153A, S14.154A, S14.155A, S14.156A, S14.157A, S14.158A, S24.101A, S24.102A,S24.103A, S24.104A, S24.111A, S24.112A, S24.113A, S24.114A, S24.131A, S24.132A, S24.133A, S24.134A, S24.151A, S24.152A, S24.153A, S24.154A |
| **Temporary neurologic dysfunction** |  |  |
| Transient ischemic attack | 435.8, 435.9 | G45.1, G45.8, G45.9, I67.848 |
| Delirium | 290.11, 290.3, 290.41, 292.81, 293, 293.1, 292.11, 292.12 | F03.90, F05, F01.51, F19.921, F06.2, F06.0, F06.30, F06.4, F06.1, F53, F19.950, F19.951 |
| **Infectious** |  |  |
| Bacteremia | 790.7 | R78.81 |
| Sepsis | 995.91, 995.92, 038.0-038.9 | A41.9, R65.20, A40.9, A41.2, A41.01, A41.02, A41.1, A40.3, A41.4, A41.89, A41.9, A41.50, A41.3, A41.51, A41.52, A41.53 |
| Wound infection | 998.51, 998.59 | T81.4XXA, K68.11 |
| infections of mediastinum | 513.1 | J85.3 |
| Pulmonary infection/pneumonia | 481, 482.0, 482.1, 482.2, 482.30, 482.31, 482.32, 482.39, 482.40, 482.41, 482.42, 482.49, 482.81, 482.82, 482.83, 482.89, 482.9, 483.0, 485, 486, 507.0, 997.31, 997.32 | J13, J18.1, J15.0, J15.1, J14, J15.4, J15.3, J15.20, J15.211, J15.212, J15.29, J15.8, J15.5, J15.6, J15.8, J15.9, J15.7, J18.0, J18.9, J69.0, J95.851, J95.89 |
| Urinary tract infection | 599.0 | N39.0 |
| Endocarditis | 421.0, 421.1, 421.9, 112.81, 364.2, 115.04, 115.14, 115.94, 424.90, 424.91, 424.99 | I33.0, I39, I33.9, B37.6, H20.819, H40.40X0, H20.23, H20.829, B39.4, I39, B39.5, B39.9, I38 |
| Pericarditis | 420.0, 420.9 | I32, I30.9, I30.0, I30.8 |
| Skin/soft tissue infection and infection of unknown site | 682, 686 | K12.2, L03.211, L03.212, L03.213, L03.211, L03.212, L03.221, L03.222, L03.319, L03.329, L03.119, L03.129, L03.317, L03.811, L03.818, L03.891, L03.898, L03.90, L03.91, L08.0, L88, L08.89, L98.0, E83.2, L08.9 |

**Supplemental Table 2.** Descriptive characteristics of healthcare facilities treating acute type A aortic dissection in two cohorts

|  | **US NIS cohort** | | **Chinese cohort** | |
| --- | --- | --- | --- | --- |
|  | N | (%) | N | (%) |
| **Hospitals, *n*** | 1115 |  | 11 |  |
| **Annual operation volume** |  |  |  |  |
| 1-9 | 752 | 67.44 | 5 | 45.45 |
| 10-49 | 359 | 32.20 | 5 | 45.45 |
| 50+ | 4 | 0.36 | 1 | 9.10 |
| **Region** |  |  |  |  |
| Northeast | 191 | 17.1 | NA |  |
| Midwest | 277 | 24.8 |  |  |
| South | 377 | 33.8 |  |  |
| West | 270 | 24.2 |  |  |
| **Location and teaching status** |  |  |  |  |
| Urban teaching | 961 | 86.2 | 11 | 100 |
| Urban non-teaching | 136 | 12.2 | 0 | 0 |
| Rural | 18 | 1.6 | 0 | 0 |
| **Ownership** |  |  |  |  |
| Government, non-federal | 119 | 10.7 | 11 | 100 |
| Private, nonprofit | 890 | 79.8 | 0 | 0 |
| Private, investor-owned | 106 | 9.5 | 0 | 0 |
| **Bed size*** |  |  |  |  |
| Large | 815 | 73.1 | 11 | 100 |
| Medium | 231 | 20.7 | 0 | 0 |
| Small | 69 | 6.2 | 0 | 0 |

Footnotes:

*For hospitals with more than one bed size categories, we considered the bed size category of the earliest occurrence as fixed variable across the study period.

Abbreviations: IQR, inter quartile range; NA, not applicable

**Supplemental Table 3.** Predictive factors associated with overall survival in Chinese cohort of type A aortic dissection

| **Characteristics** | **Crude model** | | | **Fully adjusted model** | | |
| --- | --- | --- | --- | --- | --- | --- |
|  | HR | (95% CI) | *P*-value | HR | (95% CI) | *P*-value |
| **Demographics** |  |  |  |  |  |  |
| Age | 1.03 | 1.02-1.04 | <.0001 |  |  |  |
| ≤49 | reference |  |  |  |  |  |
| 50-59 | 0.89 | 0.59-1.33 | 0.56 | 0.78 | 0.48- 1.25 | 0.30 |
| 60-69 | 1.5 | 1.03-2.20 | 0.04 | 0.95 | 0.52-1.73 | 0.86 |
| 70+ | 2.87 | 1.92-4.29 | <.0001 | 1.87 | 0.95-3.69 | 0.07 |
| Sex | 1.15 | 0.84-1.59 | 0.38 | 0.96 | 0.67-1.36 | 0.80 |
| BMI | 1.03 | 0.93-1.14 | 0.58 |  |  |  |
| **Clinical characteristics** |  |  |  |  |  |  |
| Charlson comorbidity index |  |  |  |  |  |  |
| 0-2 | reference |  |  |  |  |  |
| 3-4 | 1.79 | 1.28-2.51 | 0.0007 | 1.37 | 0.81-2.33 | 0.23 |
| 5+ | 5.8 | 4.05-8.30 | <.0001 | 2.24 | 1.09-4.62 | 0.03 |
| Type 2 diabetes | 1.7 | 1.05-2.73 | 0.03 |  |  |  |
| Hypertension | 1.44 | 0.96-2.15 | 0.08 |  |  |  |
| Dyslipidemia | 1.52 | 0.82-2.80 | 0.18 |  |  |  |
| Chronic kidney disease | 3.05 | 1.94-4.78 | <.0001 | 2.32 | 1.36-3.94 | 0.002 |
| Coronary artery disease | 1.85 | 1.15-2.98 | 0.01 |  |  |  |
| Heart failure | 2.79 | 1.97-3.96 | <.0001 | 1.75 | 1.16-2.65 | 0.007 |
| liver disease | 4.19 | 2.78-6.33 | <.0001 | 2.33 | 1.44-3.78 | 0.001 |
| Stroke | 4.07 | 2.78-5.95 | <.0001 | 2.48 | 1.64-3.76 | <.0001 |
| Total arch | reference |  |  |  |  |  |
| Ascending/hemi-arch | 0.71 | 0.47-1.08 | 0.13 | 0.6 | 0.38-0.95 | 0.03 |
| Single* | reference |  |  |  |  |  |
| Compound** | 1.46 | 1.09-1.95 | 0.01 | 1.49 | 1.08-2.06 | 0.01 |

Footnotes:

*Only total arch or ascending/ hemi-arch were used in TAAD patients

**TAAD patients were treated with total arch or ascending/hemi-arch in combination with at least one of the following operations: Aortic valve replacement, Root replacement, Aortic valvuloplasty, Coronary artery bypass grafting, Bypass surgery, Mitral valve replacement or plasty and Pacemaker implantation

**Supplemental Table 4.** Descriptive characteristics of surgical types of acute type A aortic dissection in Chinese cohorts

| **Surgical types** | | 2012-2013 | | 2014-2015 | | 2016-2017 | | 2018-2019 | | 2020-2021 | | Total | |
| --- | --- | --- | --- | --- | --- | --- | --- | --- | --- | --- | --- | --- | --- |
|  |  | N=108 | % | N=135 | % | N=162 | % | N=274 | % | N=393 | % | N=1073 | % |
| **Total arch** | | 84 | 77.8% | 114 | 84.5% | 144 | 88.9% | 225 | 82.1% | 298 | 75.9% | 865 | 80.7% |
| **Ascending/hemi-arch** | | 24 | 22.2% | 21 | 15.6% | 18 | 11.1% | 49 | 17.9% | 95 | 24.1% | 207 | 19.3% |
| **Single*** | | 54 | 50.0% | 87 | 64.4% | 97 | 59.9% | 210 | 76.6% | 272 | 69.2% | 720 | 67.2% |
| **Compound**** | | 54 | 50.0% | 48 | 35.6% | 65 | 40.1% | 64 | 23.4% | 121 | 30.8% | 352 | 32.8% |
| **Combination** | |  |  |  |  |  |  |  |  |  |  |  |  |
| Total arch | single | 47 | 43.5% | 75 | 55.6% | 87 | 53.7% | 165 | 60.2% | 207 | 52.7% | 581 | 54.2% |
|  | compound* | 37 | 34.3% | 39 | 28.9% | 57 | 35.2% | 60 | 21.9% | 91 | 23.2% | 284 | 26.5% |
| Ascending  /hemi-arch | single | 7 | 6.5% | 12 | 8.9% | 10 | 6.2% | 45 | 16.4% | 65 | 16.5% | 139 | 13.0% |
|  | compound* | 17 | 15.7% | 9 | 6.7% | 8 | 4.8% | 4 | 1.5% | 30 | 7.6% | 68 | 6.3% |

Footnotes:

*Only total arch or ascending/hemi-arch were used in TAAD patients

**TAAD patients were treated with total arch or ascending/hemi-arch in combination with at least one of the following operations: Aortic valve replacement, Root replacement, Aortic valvuloplasty, Coronary artery bypass grafting, Bypass surgery, Mitral valve replacement or plasty and Pacemaker implantation

**Supplemental Table 5.** Impact of different surgical types on outcomes in Chinese cohorts

| **Outcomes** | Ascending/  hemi-arch  N=207 | Total arch  N=865 | P value | Single*  N=720 | Compound**  N=353 | P value |
| --- | --- | --- | --- | --- | --- | --- |
| **in-hospital mortality** | 9.7 | 14.2 | 0.083 | 11.7 | 16.7 | 0.022 |
| Patients hospitalized for 1-10 days | 61.1 | 82.4 | 0.045 | 70.8 | 92.1 | 0.011 |
| Patients hospitalized for 11-20 days | 4.2 | 5.8 | 0.55 | 4.5 | 7.1 | 0.26 |
| Patients hospitalized for 21-30 days | 3.3 | 6.7 | 0.31 | 5.2 | 8.1 | 0.29 |
| Patients hospitalized for 30+ days | 9.1 | 9.3 | 0.97 | 9.6 | 8.3 | 0.80 |
| **Los, days** |  |  | 0.51 |  |  | 0.13 |
| Patients hospitalized for 1-10 days | 8.7 | 9.8 |  | 9.0 | 10.8 |  |
| Patients hospitalized for 11-20 days | 15.9 | 17.5 |  | 39.9 | 44.2 |  |
| Patients hospitalized for 21-30 days | 29.5 | 32.6 |  | 32.2 | 31.4 |  |
| Patients hospitalized for 30+ days | 45.9 | 40.1 |  | 18.9 | 13.6 |  |
| **Costs, in 1,000 US dollar^#^** |  |  | 0.0003 |  |  | 0.88 |
| Patients hospitalized for 1-10 days | 26.1 (20.3-29.7) | 30.0 (23.9-33.3) |  | 27.9 (23.0-32.6) | 31.5 (24.0-33.3) |  |
| Patients hospitalized for 11-20 days | 28.4 (24.5-32.1) | 31.7 (26.5-33.1) |  | 30.1 (26.1-33.0) | 32.9 (26.8-32.8) |  |
| Patients hospitalized for 21-30 days | 33.1 (27.8-35.1) | 35.7 (29.0-38.2) |  | 34.7 (28.5-37.2) | 36.3 (29.3-38.3) |  |
| Patients hospitalized for 30+ days | 44.9 (31.8-49.4) | 52.1 (33.6-61.0) |  | 50.7 (34.5-54.6) | 51.0 (31.6-62.8) |  |

Footnotes:

*Only total arch or ascending/hemi-arch were used in TAAD patients

**TAAD patients were treated with total arch or ascending/hemi-arch in combination with at least one of the following operations: Aortic valve replacement, Root replacement, Aortic valvuloplasty, Coronary artery bypass grafting, Bypass surgery, Mitral valve replacement or plasty and Pacemaker implantation

# Use nonparametric test

**Supplemental Figure 1.** Survival plot on long-term all-cause mortality in the Chinese cohort


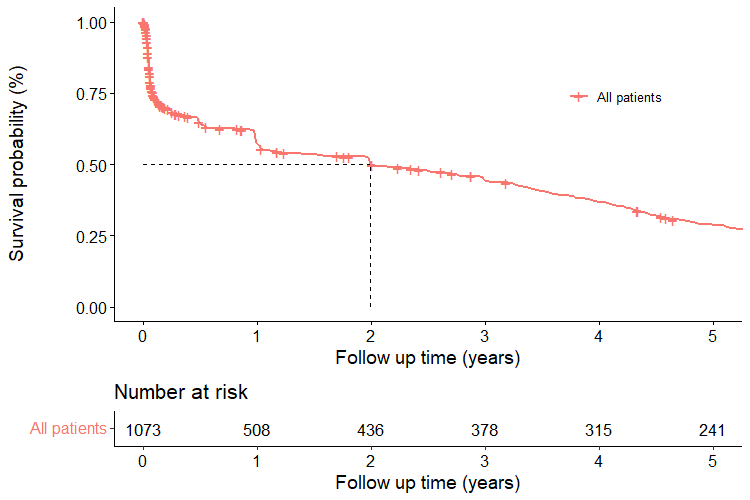


**Supplemental Figure 2.** Crude and standardized complication trend charts for the Chinese and US cohorts


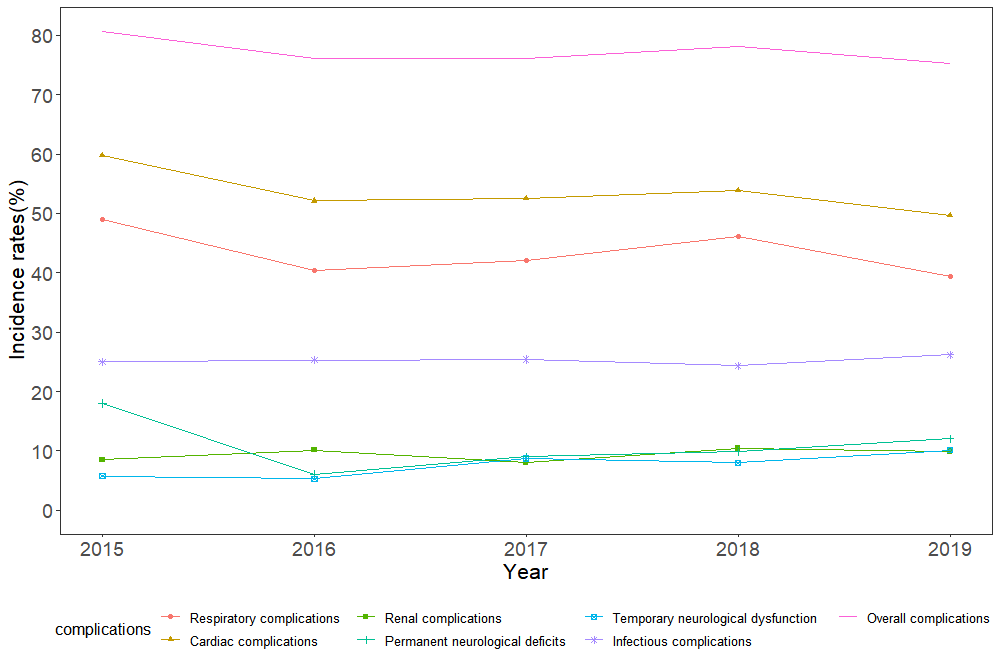


1. Crude complication trend for the US cohort


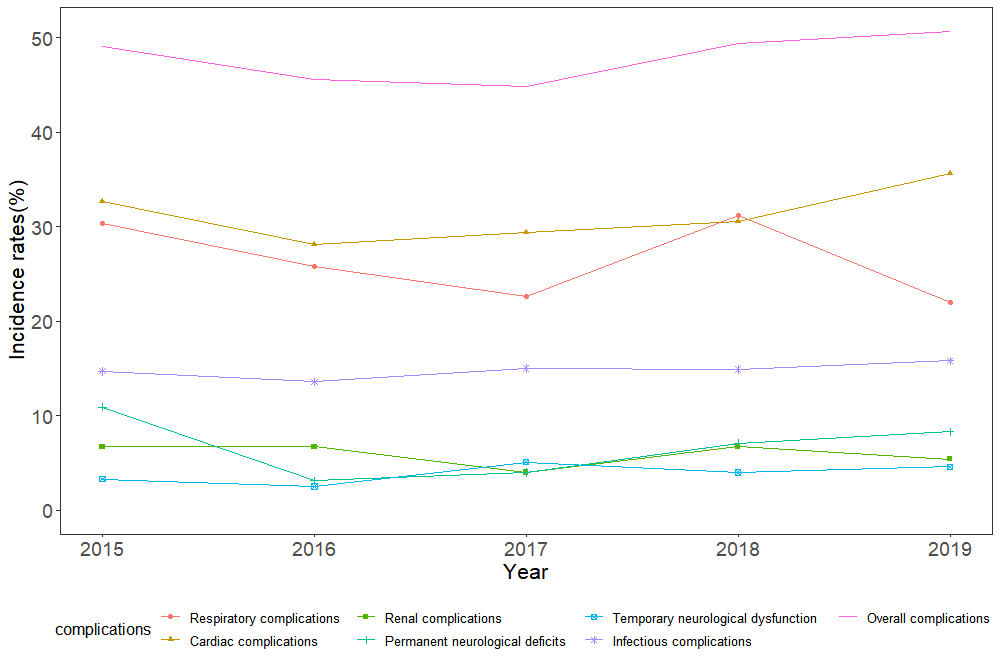


1. Standardized complication trend for the US cohort


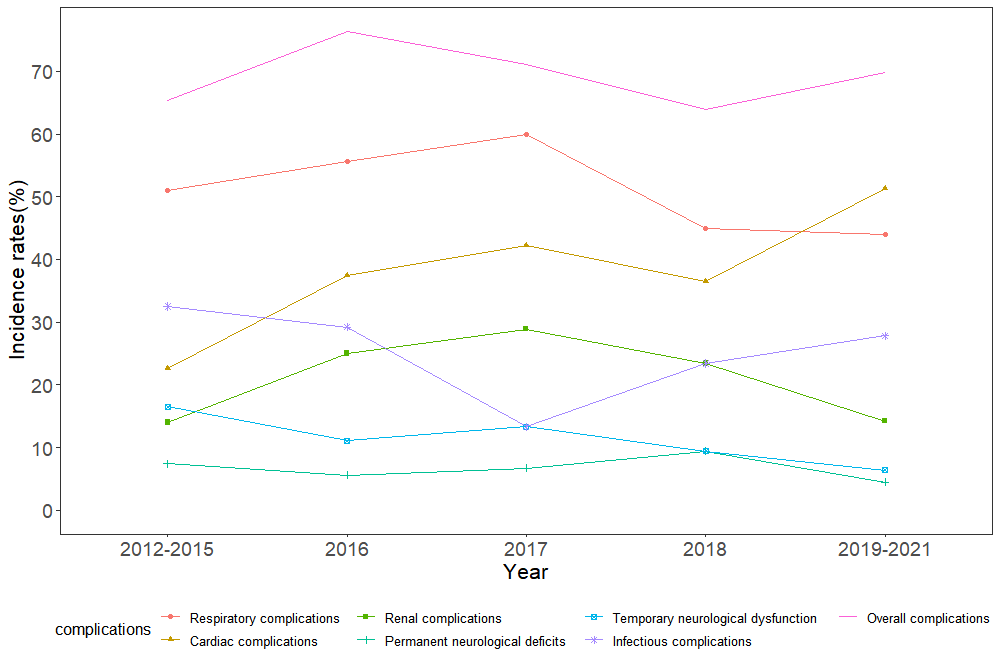


1. Crude complication trend for the Chinese cohort


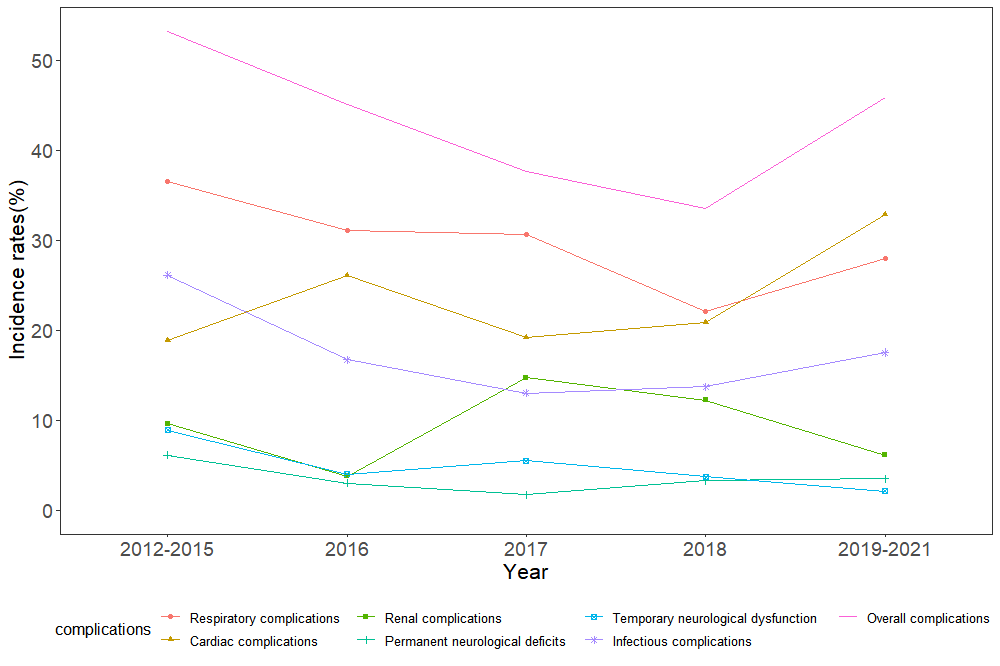


1. Standardized complication trend for the Chinese cohort

**Supplemental Figure 3**. Survival plot for subgroups of long-term all-cause mortality in the Chinese cohort


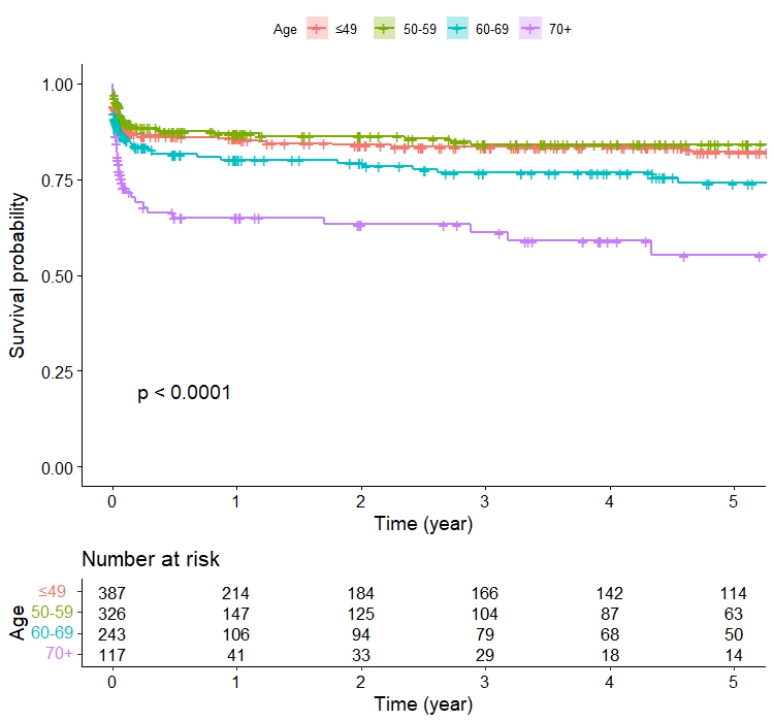


1. Age-related survival plot of long-term all-cause mortality


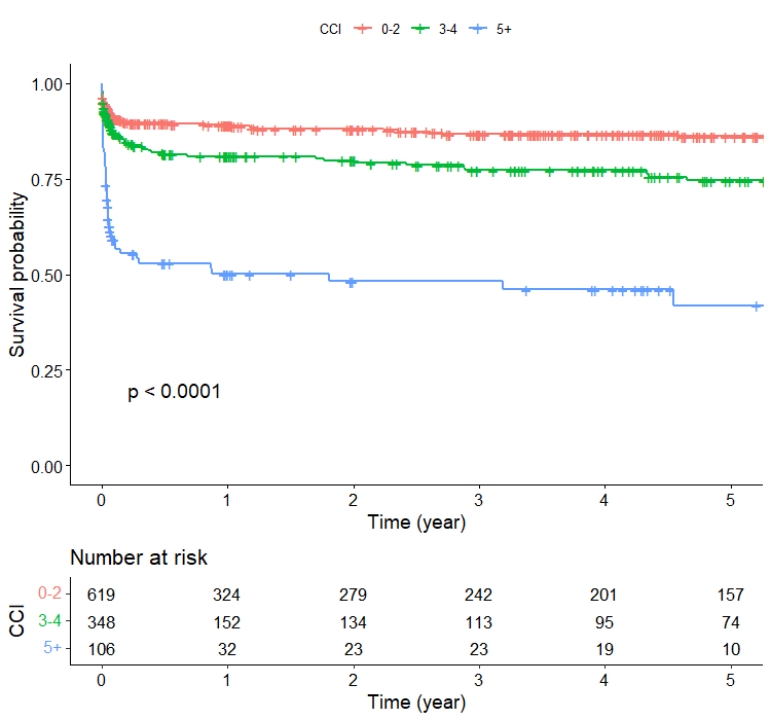


1. Charlson comorbidity index-related survival plot of long-term all-cause mortality

Abbreviations: CCI: Charlson comorbidity index.


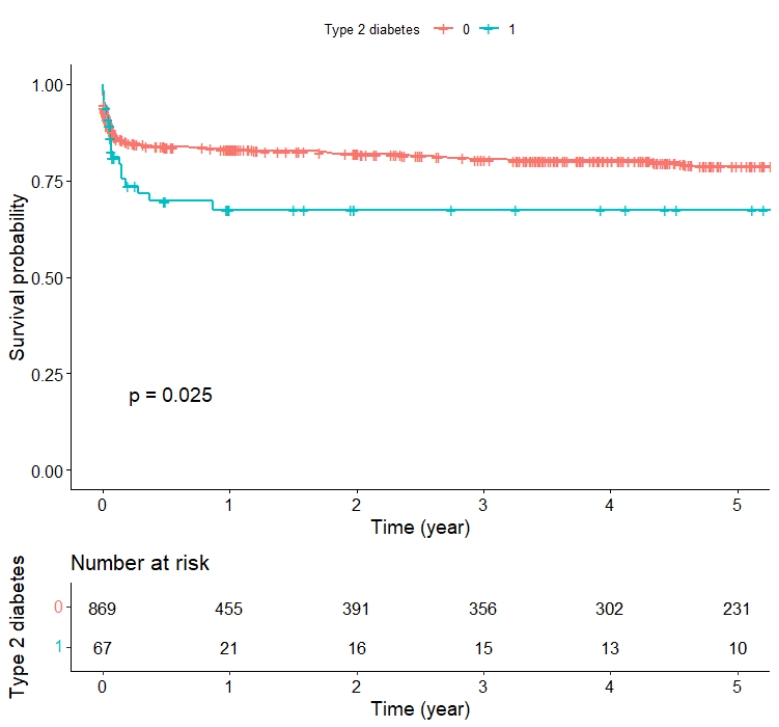


1. Type 2 diabetes-related survival plot of long-term all-cause mortality


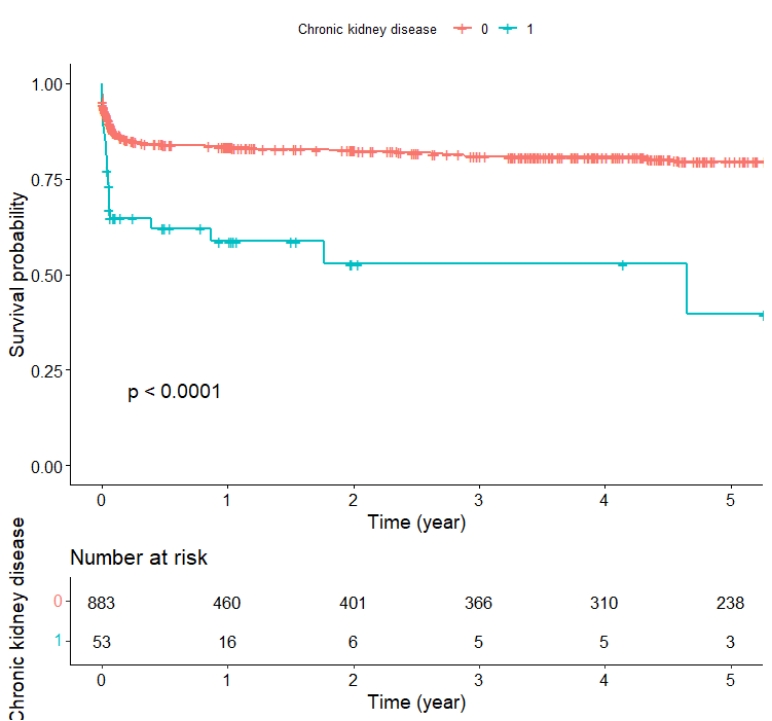


1. Chronic kidney disease-related survival plot of long-term all-cause mortality


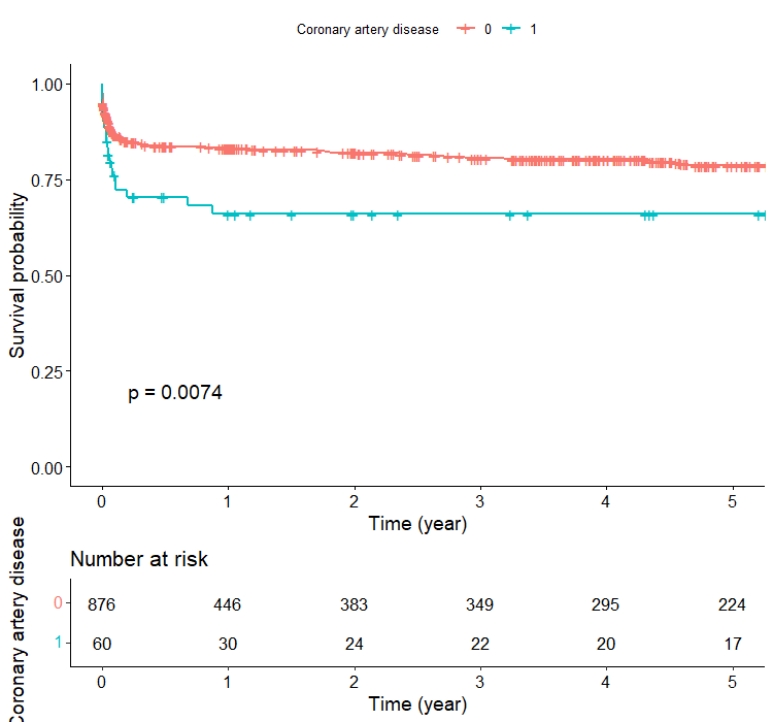


1. Chronic artery disease-related survival plot of long-term all-cause mortality
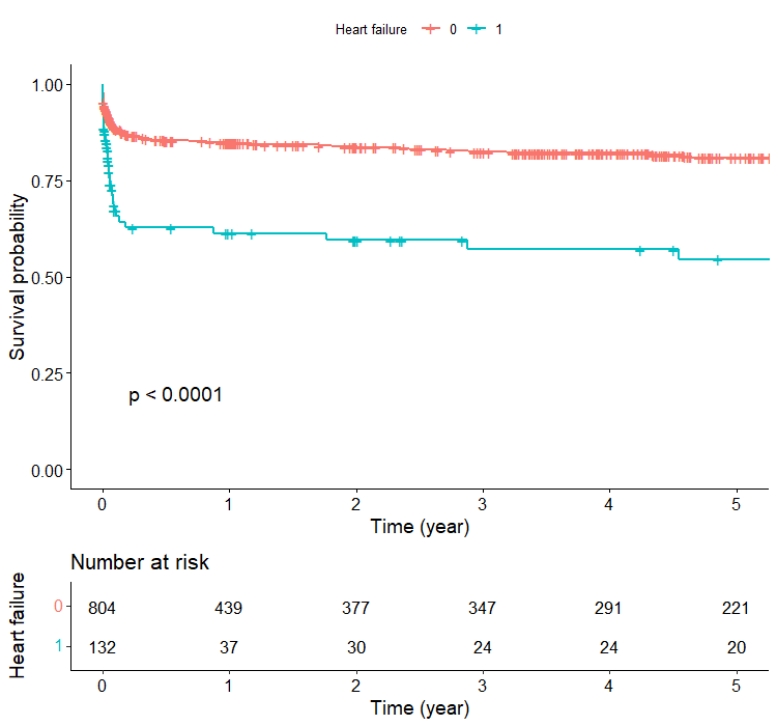

2. Heart failure-related survival plot of long-term all-cause mortality


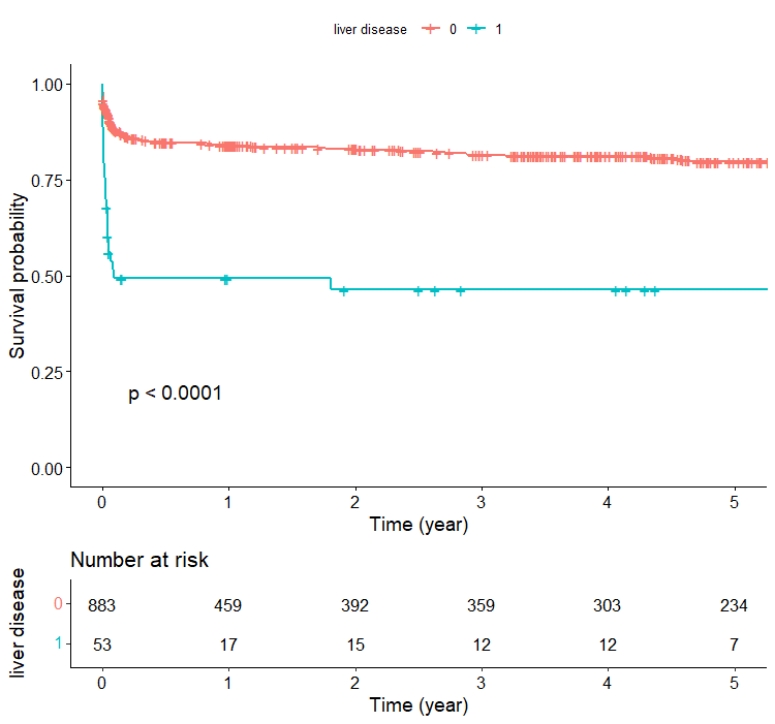


1. Liver disease-related survival plot of long-term all-cause mortality


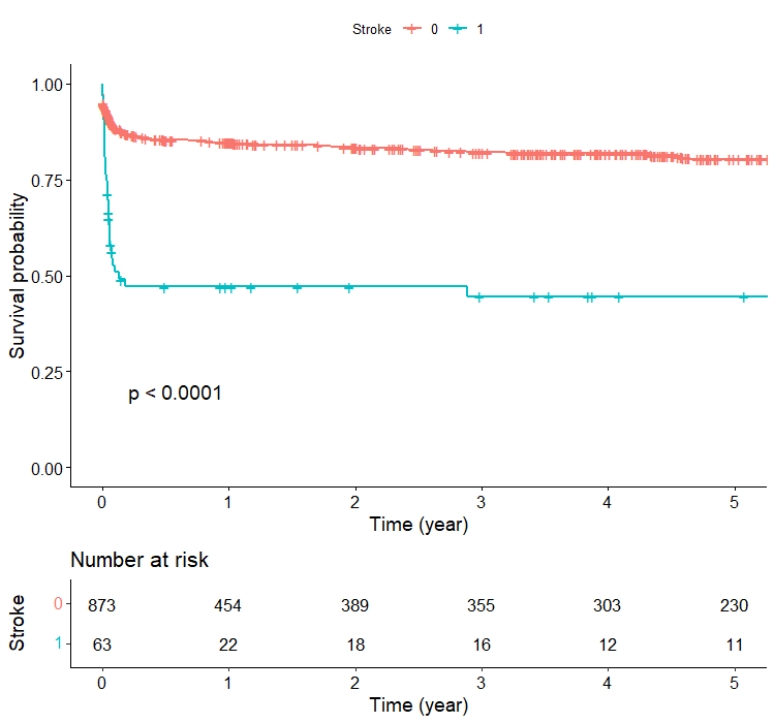


1. Stroke-related survival plot of long-term all-cause mortality


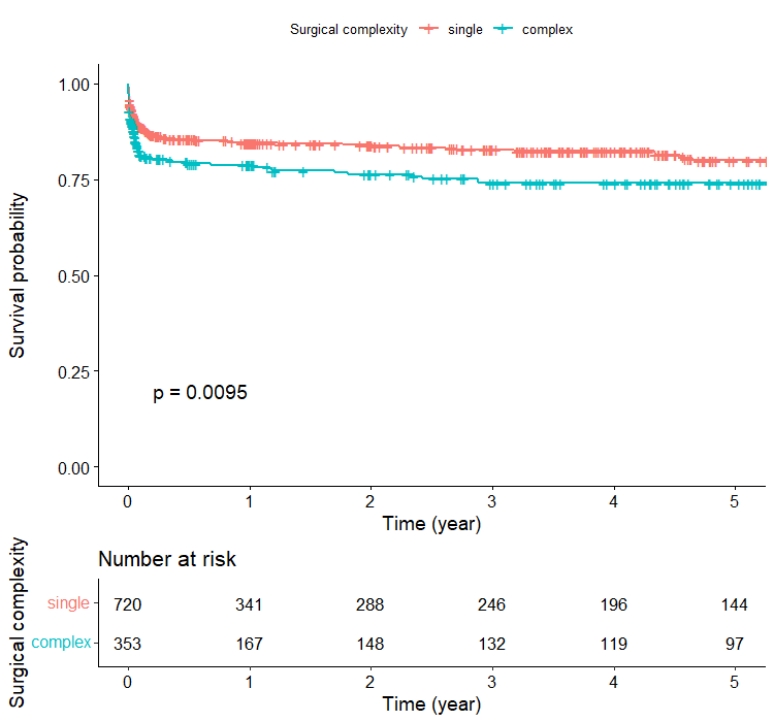


Surgical complexity-related survival plot of long-term all-cause mortality
